# A robust CD8^+^ T cell-related classifier for predicting the prognosis and efficacy of immunotherapy in stage III lung adenocarcinoma

**DOI:** 10.3389/fimmu.2022.993187

**Published:** 2022-08-31

**Authors:** Jinteng Feng, Longwen Xu, Shirong Zhang, Luying Geng, Tian Zhang, Yang Yu, Rui Yuan, Yusheng He, Zhuhui Nan, Min Lin, Hui Guo

**Affiliations:** ^1^ Department of Medical Oncology, the First Affiliated Hospital of Xi’an Jiaotong University, Xi’an, China; ^2^ Bioinspired Engineering and Biomechanics Center (BEBC), Xi’an Jiaotong University, Xi’an, China; ^3^ Key Laboratory of Biomedical Information Engineering, School of Life Science and Technology, Xi’an Jiaotong University, Ministry of Education of China (MOE), Xi’an, China; ^4^ Key Laboratory of Environment and Genes Related to Diseases, Xi’an Jiaotong University, Ministry of Education of China (MOE), Xi’an, China

**Keywords:** lung adenocarcinoma, CD8^+^ T cell, classifier, prognosis, immunotherapy

## Abstract

Patients with stage III lung adenocarcinoma (LUAD) have significant survival heterogeneity, meanwhile, CD8^+^ T cell has a remarkable function in immunotherapy. Therefore, developing novel biomarkers based on CD8^+^ T cell can help evaluate the prognosis and guide the strategy of immunotherapy for patients with stage III LUAD. Thus, we abstracted twelve datasets from multiple online databases and grouped the stage III LUAD patients into training and validation sets. We then used WGCNA and CIBERSORT, while univariate Cox analysis, LASSO analysis, and multivariate Cox analysis were performed. Subsequently, a novel CD8^+^ T cell-related classifier including HDFRP3, ARIH1, SMAD2, and UPB1 was developed, which could divide stage III LUAD patients into high- and low-risk groups with distinct survival probability in multiple cohorts (all P < 0.05). Moreover, a robust nomogram including the traditional clinical parameters and risk signature was constructed, and t-ROC, C-index, and calibration curves confirmed its powerful predictive capacity. Besides, we detected the difference in immune cell subpopulations and evaluated the potential benefits of immunotherapy between the two risk subsets. Finally, we verified the correlation between the gene expression and CD8^+^ T cells included in the model by immunohistochemistry and validated the validity of the model in a real-world cohort. Overall, we constructed a robust CD8^+^ T cell-related risk model originally which could predict the survival rates in stage III LUAD. What’s more, this model suggested that patients in the high-risk group could benefit from immunotherapy, which has significant implications for accurately predicting the effect of immunotherapy and evaluating the prognosis for patients with stage III LUAD.

## Introduction

Lung cancer has become the first reason of all cancer-associated deaths worldwide, also in China, which accounts for nearly one million deaths each year (1), and approximately 85% cases are Non-small cell lung cancer (NSCLC) (2). What’s more, lung adenocarcinoma (LUAD) represents the most common pathological subtype in NSCLC, for which not been found specific risk factors (3). Besides, statistics show that almost 30% of NSCLC patients are diagnosed with locoregionally or locally advanced disease, which is stage III (4). Although there has shaped a comprehensive therapy pattern including surgery, chemotherapy, radiotherapy, targeted therapy, and immunotherapy in recent years, the survival rate is not satisfactory especially for locally advanced LUAD (5), despite the absence of metastases. According to the tumor, node, metastasis (TNM) staging (8th edition), stage III is subclassified into stage IIIA, IIIB, and IIIC (6). For this heterogeneous group which presents a wide spectrum of clinical features including multiple statuses of lymph nodes metastasis, their 5-year overall survival (OS) rates are totally different (7). Hence, precisely distinguishing and predicting the prognosis of each subtype of stage III LUAD patients would help to formulate accurate treatment and improve the survival rate.

Immunotherapy has recently shown great efficacy for patients with stage III unresectable NSCLC, especially for those trapped in the lack of targetable mutations, who could not benefit from targeted therapy such as tyrosine kinase inhibitors (TKIs) (8, 9). As the most iconic treatment of immunotherapy, immune checkpoint blockades (ICBs) have established a solid position and could be chosen as the first-line treatment (10). Among them, monoclonal antibodies against programmed death 1(PD-1) and its ligand (PD-L1) are the most widely used ICBs in locally advanced LUAD at present, which has shown obvious survival benefits compared to traditional chemotherapy (11). Nonetheless, only canonical biomarkers like PD-L1 and tumor mutational burden (TMB) are used in clinical practice, which also has their own limitations (12). Therefore, the significance of identifying novel immune-related biomarkers is highlighted, which may help to select those patients who are most likely to benefit from ICBs.

As the indispensable part of cancer, the tumor microenvironment (TME) is essential nature in cancer progression. Alternatively, a variety of immune cell types within TME drive a fundamental environment that could respond to immunotherapy (13). Particularly, among all immune cell types, the CD8^+^ T cell is the most important conductor in the cancer-immunity cycle and its activation and infiltration play a crucial role in immunotherapy (14). However, some co-inhibitory molecules or receptors in the TME causing T cell exhaustion might impair their potential to fight cancer cells (15). Therefore, how to find out and confirming the biomarkers correlated to CD8^+^ T cell become necessary. In recent years, some works have revealed how a single intrinsic gene of LUAD cells influences CD8^+^ T cells in TME. For instance, knockdown of GBE1 could increase recruitment of CD8^+^ T lymphocytes (16), TP53-deficient LUAD cells promoted CD8^+^ T cells exhaustion (17), and high expression of MSH2 correlated with increased CD8^+^ T cells infiltration (18), whereas, these genes cannot demonstrate the whole signature and predict the various prognosis of LUAD patients. Besides, based on immune-related genes, researchers have established a few prognostic models to make predictions for the survival risk (19, 20). Nevertheless, we need to exploit a novel model containing multiple biomarkers about the heterogenous locally advanced LUAD based on as many databases as possible, which is comprehensive enough to reach a satisfying prognostic value and predict the immunotherapy response.

Based on the rapid development of bioinformatics, in this study, we aim to establish a reliable CD8^+^ T cell-related signature to estimate the prognostic stratification and the effect of immunotherapy in locally advanced LUAD. First, we integrated datasets about stage III LUAD, which were from multiple online databases. To identify the hub CD8^+^ T cell-related biomarkers, we then used weighted gene co-expression network analysis (WGCNA). Subsequently, we developed a novel CD8^+^ T cell-related classifier and constructed a robust nomogram to predict survival probability. Besides, the predictive performance was further validated in the multiple test sets. Moreover, we detected the difference in immune cell subpopulations and evaluated the potential benefits of immunotherapy between the two risk subsets. Finally, the valuation of this model was verified in a real-world cohort in evaluating immunotherapy efficacy. Taken together, it was expected that this CD8^+^ T cell-related model could contribute to predicting survival rates and accurately working out therapeutic strategies for locally advanced LUAD patients.

## Materials and methods

### Study population, gene expression data, and processing

The gene expression profiles and clinical parameters of primary LUAD patients from 12 public cohorts were retrospectively analyzed, including 11 microarray datasets from the Gene Expression Omnibus (GEO) and Array-Express, and 1 RNA-Seq expression profile from The Cancer Genome Atlas (TCGA). Only patients who meet the following two criteria were included: i) detailed TNM staging information includes stage IIIA and IIIB according to the 7th edition of TNM classification of malignant tumors; ii) OS information includes follow-up time and survival status. The Combat algorithm was used to eliminate the batch effects. Then, the whole set was divided into training and internal validation cohorts in a ratio of 1:1 using stratified random sampling by caret R package. Another series from TCGA was used as the external validation cohort. The studies obtained from each of the databases are summarized together with series ID in **Supplementary Table S1**.

### Evaluation of tumor-infiltrating immune cells

We performed CIBERSORTx (https://cibersortx.stanford.edu/) to investigate the levels of 22 TIICs using the mRNA expression data of the training cohort. This online tool utilizes a deconvolution method to impute gene expression profiles and estimate the type and fractions of immune cells.

### Establishing the co-expression network

We used the R package **“**WGCNA**”** (21) to construct a weight co-expression network with the 7922 gene expression values in the training cohort. The levels of 22 immune-infiltrating cells were used as sample traits. When the index of scale-free topologies was set as 0.90, a scaleless network was successfully built with an optimal soft threshold power (β = 5). Next, we divided genes with similar expression patterns into the same module (minimum size = 50) using the **“**dynamic tree cutting**”** algorithm. In addition, to select the remarkable modules, Pearson**’**s test was used to evaluate the relationship between the module eigengenes and the level of the 22 types of immune cells. At last, the **“**CD8^+^ T cells**”** subtype was chosen and further study on the CD8^+^ T cell-related module was conducted.

### Pathway and process enrichment analysis

To determine the function of genes in the identified hub module, we employed the web tool **“**Metascape**”** (http://metascape.org) for pathway and process enrichment analysis (22). The tool displays the first 20 enriched terms as a bar graph. To further explore the relationship between these terms, terms with similarities greater than 0.3 are connected by edges and presented as a network graph.

### Construction and validation of the risk model based on CD8^+^ T cell-related genes

Univariate Cox regression analysis was performed to estimate the hazard proportions for genes of the highest correlation with CD8^+^ T cells (yellow module). Then, to further screen the prognosis of CD8^+^ T cell-related genes with the best predictive performance, the **“**glmnet**”** R package (23) was used to perform the LASSO regression analysis with ten-fold cross-validation. Next, based on the AIC (Akaike information criterion) value on the prognosis CD8^+^ T cell-related genes, the bi-directional stepwise multivariate Cox regression was used for choosing the ones that minimize the AIC to obtain the best model fit. A prognostic CD8^+^ T cell-related risk score model of stage III LUAD patients was then established based on combining the multiplication of the multivariate Cox regression coefficient by its corresponding normalized mRNA expression value. The risk score= ∑(the multivariate Cox coefficient of CD8^+^ T cell-related genes × matching normalized expression level of these genes). We computed risk scores of each stage III LUAD patient and then divided them into high- and low-risk subsets according to the cutoff value of 28.401 determined *via* receiver operating characteristic (ROC) curve analysis using the R package **“**survminer**”**. Next, the Kaplan-Meier (KM) curve was performed to estimate the disparity in OS between low- and high-risk subsets by log-rank test. The prognostic ability of the CD8^+^ T cell-related classifier was explored with an analysis of the concordance index (C-index) and ROC curve. Then, we also used similar methods to verify the prognostic performance of the classifier constructed by the training cohort in the internal validation, external validation, and pooled validation cohorts.

Furthermore, based on univariate Cox regression and multivariate Cox regression analyses, we further confirmed whether the predictive performance of the CD8^+^ T cell-related classifier could be an independent prognostic factor compared with other clinic factors for stage III LUAD patients in multiple cohorts. At last, risk score and three traditional clinical factors were used to generate the nomogram by using **“**rms,**” “**foreign,**”** and **“**survival**”** R packages. C-index, time-dependent ROC (t-ROC) curve and calibration plots of the nomogram for 1-, 3-, and 5-year OS plots were applied to elucidate the accuracy of actual observed rates with the predicted survival probability. The **“**timeROC**”** R package was utilized to perform the t-ROC analyses.

### Prediction for response to immunotherapy or chemo-agents

Tumor immune dysfunction and exclusion (TIDE) algorithms (24) and subclass mapping (25) were used to predict clinical response to immune checkpoints between the two risk subsets in the TCGA dataset, also named the external validation set. The chemotherapy response was predicted by employing the R package **“**pRRophetic version 0.5**”** to compute the half-maximal inhibitory concentration (IC50) of four common chemo-agents (cisplatin, gemcitabine, paclitaxel, and docetaxel) in the training set (26, 27). The comparison of IC50 of these agents between groups was performed using Wilcoxon rank-sum test.

### Efficacy evaluation of immunotherapy and immunohistochemical verification for a real-world cohort

Clinicopathological features and samples of stage III NSCLC patients were collected from January 2019 to December 2021 who received immunotherapy. After the screening by exclusion criteria, twenty-eight patients were enrolled as a validation cohort from real-world for analysis (**Supplementary Figure S1**; **Supplementary Table S2**). For the evaluation of the immunotherapy efficacy, we used the Best overall response (BOR), which was defined as the best response during immunotherapy and was accessed according to RECIST1.1 (28). What’s more, all patients were followed up until May 2022, and this study was approved by the Research Ethics Committees of the First Affiliated Hospital of Xi’an Jiaotong University.

The Immunohistochemistry (IHC) was performed with a three-step method. After the dewaxing and hydrating, the tissue sections were boiled in autoclaved citric acid buffer (pH 6.0) for 20 min for antigen retrieval, and the peroxidase activity was quenched with 3% hydrogen peroxide for 15 min to avoid non-specific staining. Then, the sections were blocked for 15min followed by incubation overnight with CD8 antibody (Invitrogen, PA5-88265 at 1/100 dilution), anti-UPB1 antibody (Abcam, ab157195 at 1/100 dilution), HDGFRP3 antibody (proteintech, 12380-1-AP at 1/50 dilution), SMAD2 antibody (proteintech, 12570-1-AP at 1/500 dilution), or ARIH1 antibody (Santa, sc-390763 at 1/50 dilution) at 4°C. After that step, the sections were incubated with the secondary antibody at 37°C for 20 min. Subsequently, This step was followed by incubating with Horseradish Peroxidase for 20 min, and staining with 3,3- diaminobenzidine. At last, the sections were dehydrated and sealed after re-dyeing with hematoxylin. The IHC assays were performed by integral optical density (IOD) using Image J (29).

### Statistical analysis

Software R (version 4.1.0) and GraphPad Prism (version 8.0.0) were applied to all data analyses. The Wilcoxon test and chi-square test were performed to assess the relationship between the risk score and clinical features. Survival analysis was utilized by the KM log-rank test. In the results of the CIBERSORT method, samples with *P* < 0.05 were retained for the next analysis. Two-tailed *P* < 0.05 was considered statistical significance.

## Results

### Gene expression profile database selection according to enrollment criteria

**Figure 1:**
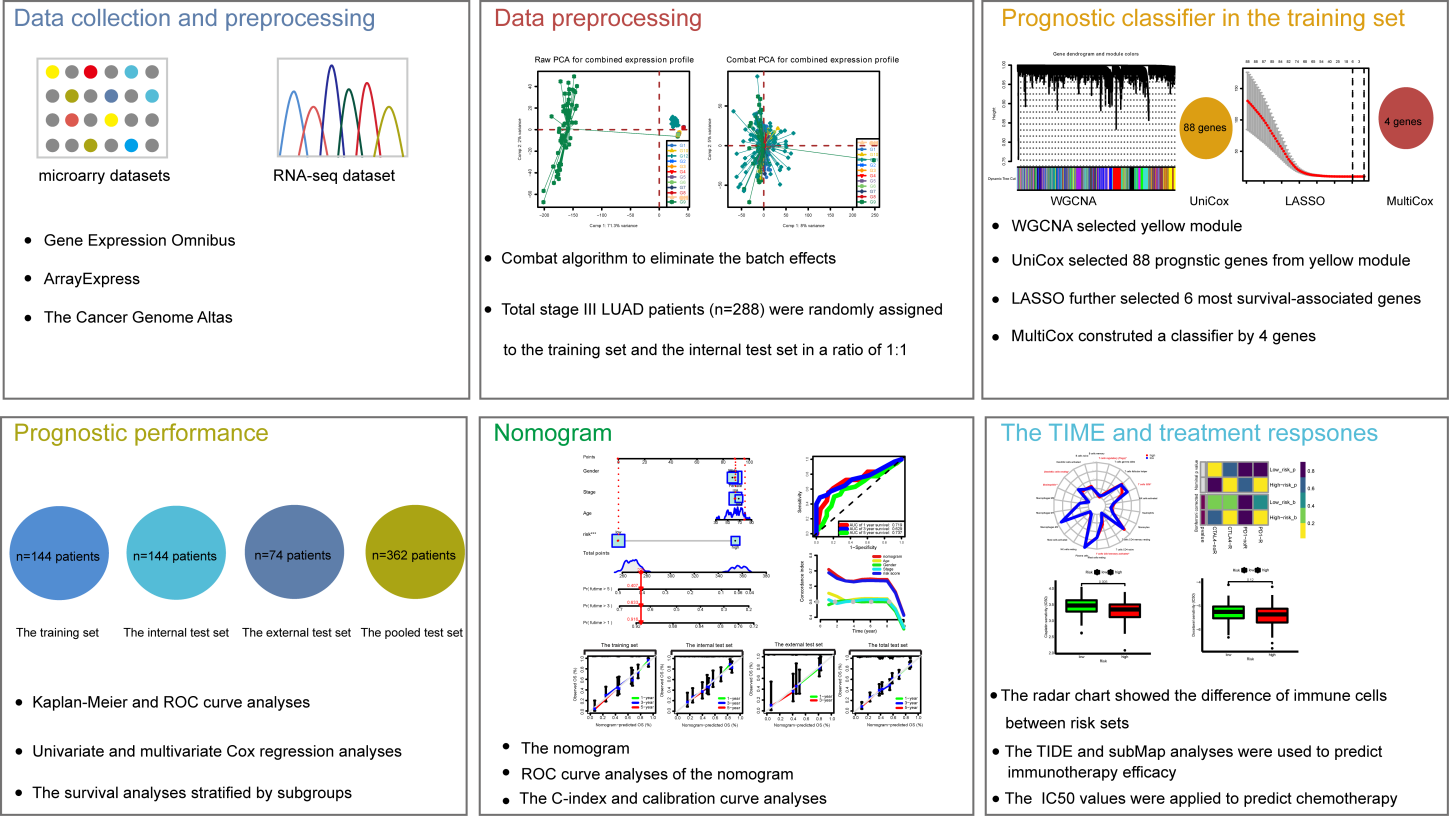
The workflow of the study design.

The study workflow design was depicted in **Figure 1**. As mentioned above, 12 series (288 LUAD patients in total) were selected. To combine these datasets, a combat method was first performed to eliminate batch effects, and the results before and after the batch correction were displayed by PCA plots, respectively **(** **Supplementary Figure S2**). Consequently, a merged cohort was integrated. To improve the precision and accuracy of the prognostic model, the 288 samples from the merged cohort were divided into training (n = 144) and internal validation (n = 144) sets in a ratio of 1:1 using stratified random sampling. Besides, the 74 patients from TCGA were employed as the external validation set, and a pooled set integrating the training, internal validation, and external validation sets was constructed.

### Identification of hub modules by WGCNA and enrichment analysis

To identify key modules correlated with CD8^+^ T cells, the mRNA gene expression profiles for 144 LUAD samples from the training cohort were extracted. Subsequently, for these LUAD samples, the different cell subtypes’ abundance was calculated by the CIBERSORT algorithm, in which seven subtypes of T cell fractions were defined as trait data for WGCNA analysis. Next, to construct the gene co-expression network of LUAD, the expression profiles of the 7921 genes were utilized. To ensure the network was scale-free, β = 5 (scale-free R^2^ = 0.9) was selected **(** **Supplementary Figures S3A** **, B** **)**. Besides, the samples of the training cohort were clustered by the average linkage and Pearson’s correlation values. Finally, a total of 18 modules were constructed by building a hierarchical clustering tree, where the gene set was independent as the tree branch. **(** **Supplementary Figure S3C** **)**.

According to the criteria of the hybrid dynamic tree cut, we got that the yellow module was significantly associated with T cells, such as CD8^+^ T cells (R^2^ = 0.25, *P* = 0.002) **(** **Supplementary Figure S4A** **)**. To elucidate the potential function and mechanism of CD8^+^ T cells, we picked the yellow module as a hub module. Additionally, we got that these genes from the hub yellow module were mainly enriched in ubiquitin protein ligase binding, SMAD binding, and lymphocyte activation after GO and KEGG enrichment analysis **(** **Supplementary Figures S4B, C** **)**.

### Establishment of the prognostic CD8^+^ T cell risk score in the training set

**Figure 2:**
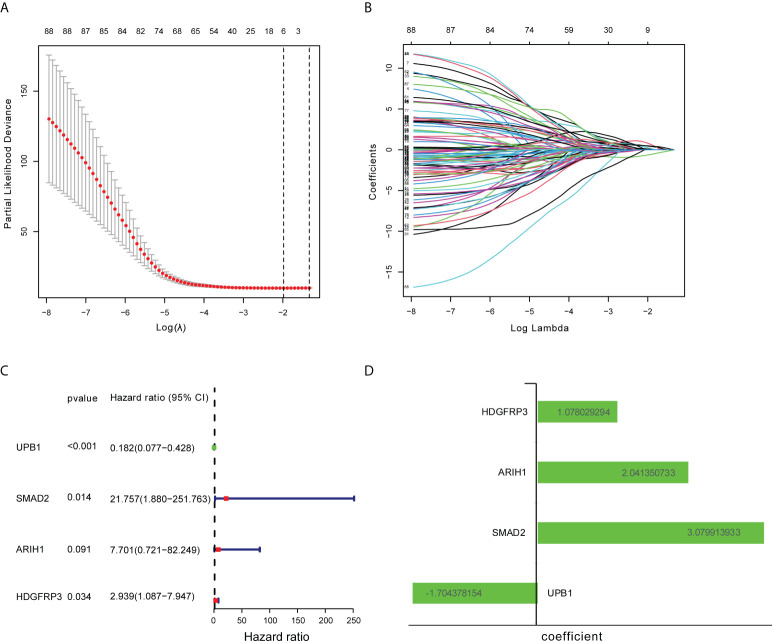
Construction of CD8^+^ T cell-related genes signature. **(A)** Ten-fold cross-validation with minimum criteria for tuning parameter selection (λ) in the LASSO model. **(B)** LASSO coefficients profiled the CD8^+^ T cell-related genes. **(C)** Multivariable Cox regression analysis of these CD8^+^ T cell-related genes adopted in the signature. **(D)** The coefficient of these CD8^+^ T cell-related genes using multivariable Cox regression analysis.

**Figure 3:**
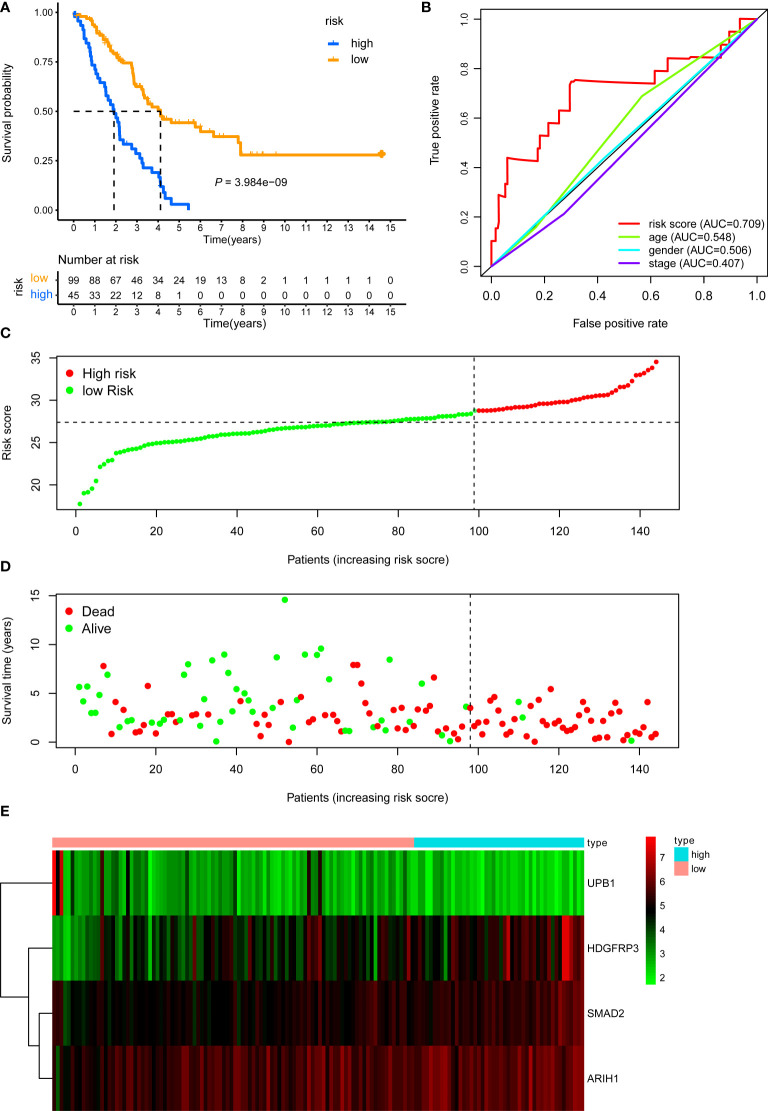
KM, t-ROC and distribution analysis of the CD8^+^ T cell-related risk score model in the training validation set. **(A)** KM curve of the CD8^+^ T cell-related signature for OS. **(B)** ROC analysis of the CD8^+^ T cell-related signature for 5-year OS. **(C)** The risk plot showed the risk score in the low-risk and high-risk groups. **(D)** The survival plot showed different survival statuses between the two risk groups. The dotted line indicates the cutoff value. **(E)** The heatmap exhibited gene expression levels between two risk groups.

There were 805 hub genes within the yellow module selected for further analysis. After univariate Cox regression analysis on these hub genes, 88 significantly prognosis-associated CD8^+^ T cell-related genes were identified in the training cohort. Then these significant genes entered LASSO COX regression analyses **(** **Figures 2A, B** **)** and multivariate Cox proportional risk regression analysis **(** **Figure 2C** **)**. Based on these analyses, the prognostic CD8^+^ risk model was constructed including the four most potential prognosis-related genes (HDFRP3, ARIH1, SMAD2, and UPB1). The risk score = (1.078 × expression level of HDGFRP3+2.041 × expression level of ARIH1+3.079 × expression level of SMAD2-1.704 × expression level of UPB1) **(** **Figure 2D** **)**. Subsequently, all LUAD patients in the training cohort were then separated into low- and high-risk groups according to the cutoff value (28.401) **(** **Figure 3C** **)**. KM survival analysis showed that patients in the high-risk group were associated with a relatively poor OS than those in the low-risk group **(**log-rank *P* = 3.984e-09, **Figure 3A** **)**, while the heatmap and survival plot showed four prognostic expression profiles and survival status between two risk groups **(** **Figures 3D, E** **)**. Besides, univariate Cox regression analysis and multivariate Cox regression analysis demonstrated that the risk score could independently predict OS after adjusting for various clinicopathologic parameters in the training cohort **(** **Table 1** **)**. Moreover, ROC analysis of 5-year OS was applied to examine the predictive capacity of the CD8^+^ risk model, thus we got the 5-year AUC of risk model was 0.709, which was markedly higher than that of age (AUC = 0.548), gender (AUC = 0.506), and stage (AUC = 0.407), indicating that it had a more robust prediction of clinical outcome than the other clinical parameters **(** **Figure 3B** **)**.

**Table 1:** Cox regression analysis in each set.

Variables	Univariate analysis		Multivariate analysis	
	HR (95% CI)	*P* value	HR (95% CI)	*P* value
**Training set (n = 144)**				
Age (<65/≥65)	1.160 (0.745-1.804)	0.511	1.190 (0.758-1.888)	0.449
Gender (female/male)	1.332 (0.853-2.080)	0.208	1.108 (0.701-1.749)	0.661
Stage (IIIA/IIIB)	0.780(0.461-1.319)	0.354	1.224 (0.697-2.150)	0.482
Risk score (low/high)	3.828 (2.390- 6.132)	2.359E-08	4.017 (2.422- 6.663)	7.154E-08
**Internal Validation set (n = 144)**				
Age (<65/≥65)	1.010 (0.761-1.341)	0.945	1.193 (0.852-1.671)	0.304
Gender (female/male)	1.226 (0.814-1.845)	0.329	1.322 (0.868-2.012)	0.193
Stage (IIIA/IIIB)	0.829 (0.513-1.339)	0.443	0.781 (0.451-1.353)	0.379
Risk score (low/high)	1.184 (1.083-1.295)	0.000209	1.197 (1.090-1.313)	0.000156
**external Validation set (n = 74)**				
Age (<65/≥65)	1.412 (0.738-2.701)	0.297	1.014 (0.503-2.044)	0.969
Gender (female/male)	1.270 (0.679-2.372)	0.454	1.157 (0.599-2.233)	0.663
Stage (IIIA/IIIB)	0.972 (0.805-2.786)	0.946	0.728 (0.274-1.928)	0.522
Risk score (low/high)	3.210 (1.670-6.171)	0.000471	3.406 (1.719-6.747)	0.000442
**pooled set (n = 362)**				
Age (<65/≥65)	1.290 (0.975-1.707)	0.075	1.358 (1.023-1.804)	0.034
Gender (female/male)	1.288 (0.976- 1.701)	0.074	1.225 (0.925-1.623)	0.157
Stage (IIIA/IIIB)	0.832 (0.584- 1.186)	0.310	1.016 (0.707-1.461)	0.930
Risk score (low/high)	2.783 (2.085- 3.714)	3.648E-12	2.858 (2.125-3.843)	3.668E-12

### Testing the signature in the internal validation set, external validation set, and the pooled set

**Figure 4:**
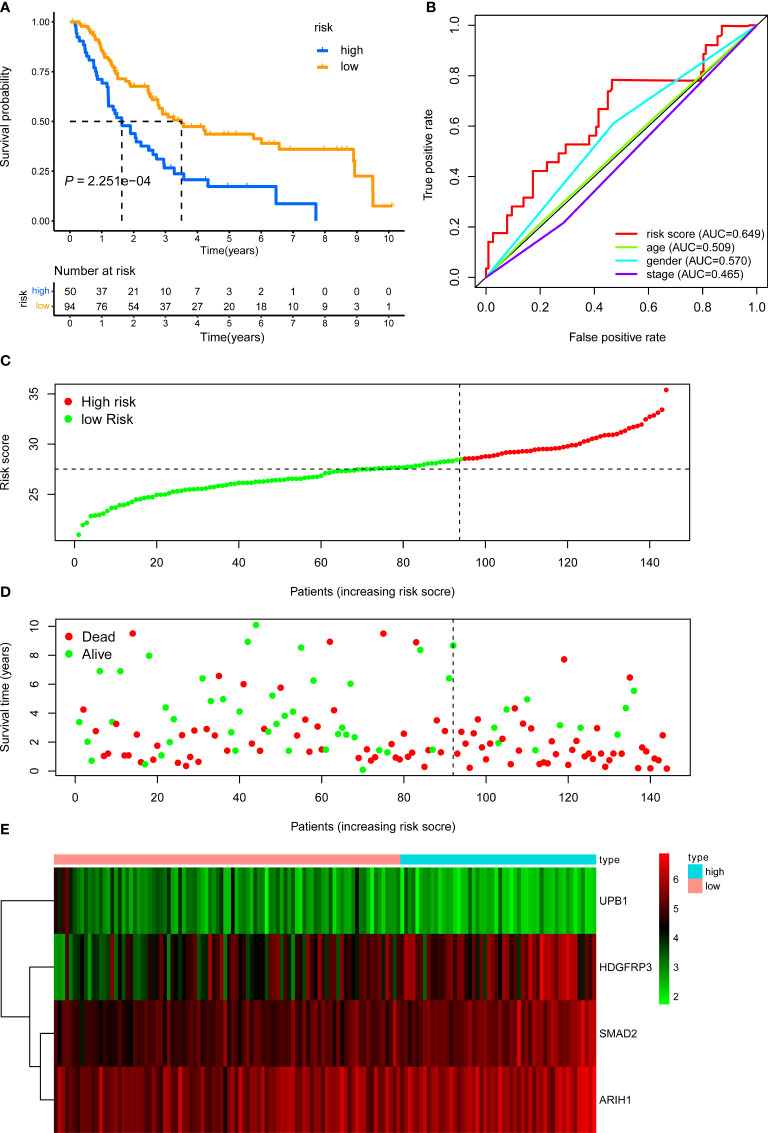
KM, t-ROC and distribution analysis of the CD8^+^ T cell-related risk score model in the internal validation set. **(A)** KM curve of the CD8^+^ T cell-related signature for OS. **(B)** ROC analysis of the CD8^+^ T cell-related signature for 5-year OS. **(C)** The risk plot showed the risk score in the low-risk and high-risk groups. **(D)** The survival plot showed different survival statuses between the two risk groups. The dotted line indicates the cutoff value. **(E)** The heatmap exhibited gene expression levels between two risk groups.

The internal validation dataset, the external validation dataset, and the pooled dataset were used to predict OS and demonstrate the predictive capacity of the risk model. The risk score in each LUAD stage III patient from the internal validation cohort was calculated based on the formula. Then, we divided the internal validation cohort into a high-risk group (n = 50) and a low-risk group (n = 94) depending on the optimal risk cutoff value in the training cohort **(** **Figure 4C** **)**. KM analysis indicated that patients in the high-risk group had a poorer prognosis compared to those in the low-risk group (log-rank *P* = 2.251e-04, **Figure 4A**). The expression profile of these four genes within our signature and survival status between two risk groups was visualized in **Figures 4D, E**. Moreover, The ROC curves for 5-year overall survival indicated that the risk score has the best predictive capacity of OS (AUC = 0.649) among the clinical parameters (**Figure 4B**).

**Figure 5:**
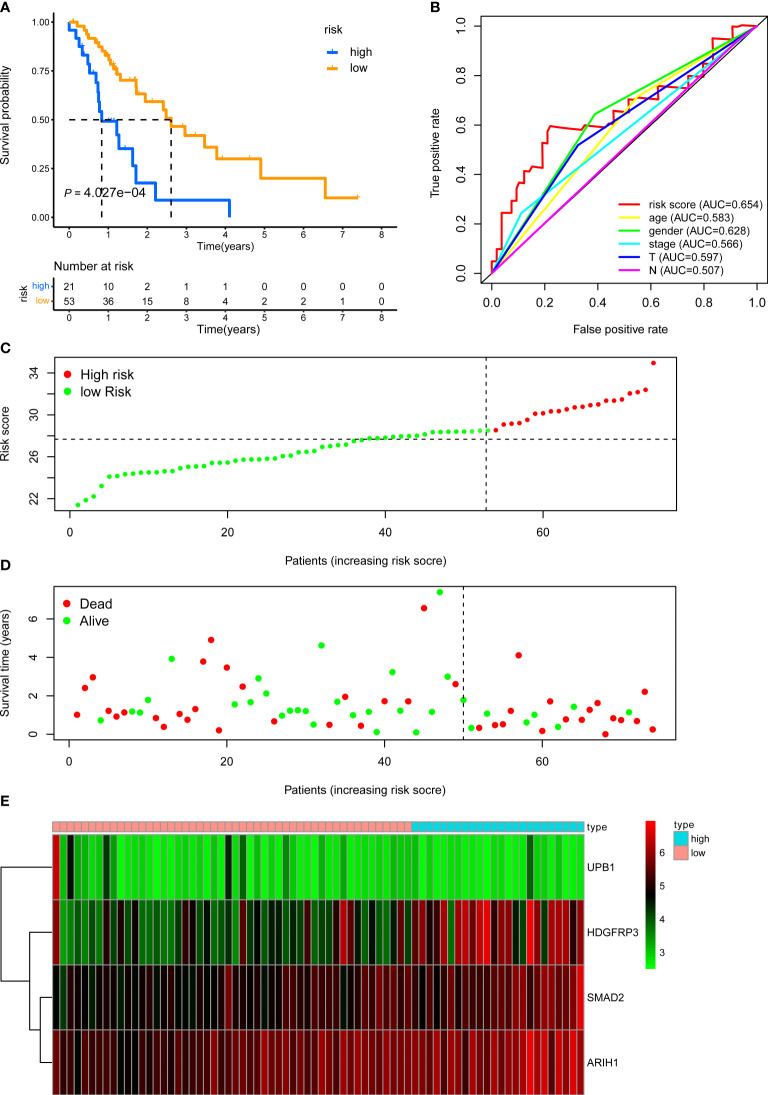
KM, t-ROC and distribution analysis of the CD8^+^ T cell-related risk score model in the external validation set. **(A)** KM curve of the CD8^+^ T cell-related signature for OS. **(B)** ROC analysis of the CD8^+^ T cell-related signature for 5-year OS. **(C)** The risk plot showed the risk score in the low-risk and high-risk groups. **(D)** The survival plot showed different survival statuses between the two risk groups. The dotted line indicates the cutoff value. **(E)** The heatmap exhibited gene expression levels between two risk groups.

We next demonstrated the prognostic predictive capacity of the CD8^+^ T cell-related classifier in the external validation dataset. The optimal risk cutoff value in the training cohort was adopted to separate the external dataset into a high-risk group (n = 21) and a low-risk group (n = 53) **(** **Figure 5C** **)**. KM analysis also revealed that high-risk patients had a poorer prognosis than those in the low-risk group (log-rank *P* value = 4.027e-04, **Figure 5A**). Besides, **Figures 5D, E** showed the expression profiles of these four genes and the survival status between the two risk groups. The ROC curves for 5-year OS also revealed that the risk score has the best predictive power of OS (AUC = 0.654) than the other traditional clinical parameters **(** **Figure 5B** **)**.

**Figure 6:**
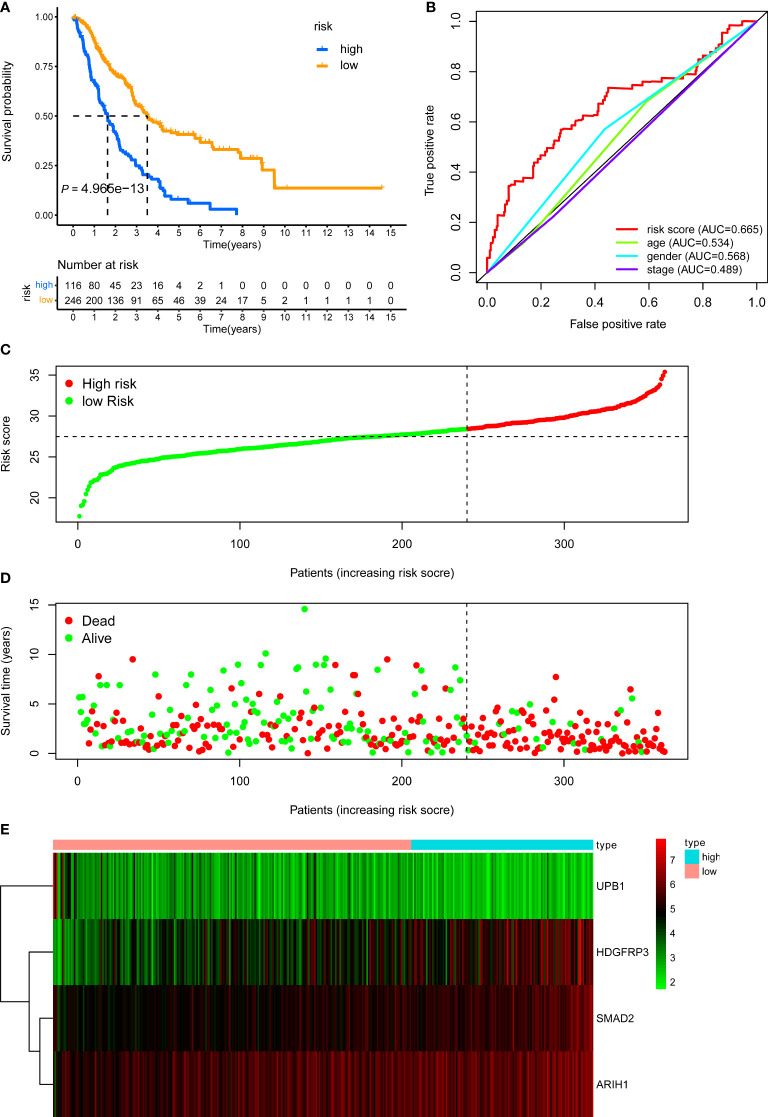
KM, t-ROC and distribution analysis of the CD8^+^ T cell-related risk score model in the pooled validation set. **(A)** KM curve of the CD8^+^ T cell-related signature for OS. **(B)** ROC analysis of the CD8^+^ T cell-related signature for 5-year OS. **(C)** The risk plot showed the risk score in the low-risk and high-risk groups. **(D)** The survival plot showed different survival statuses between the two risk groups. The dotted line indicates the cutoff value. **(E)** The heatmap exhibited gene expression levels between two risk groups.

Last, we further demonstrated the prognostic predictive capacity of the CD8^+^ T cell-related classifier in the pooled validation dataset using the same methods. The external dataset was separated into a high-risk group (n = 116) and a low-risk group (n = 246) **(** **Figure 6C** **)**. KM analysis still revealed that high-risk patients had a poorer prognosis than those in the low-risk group (log-rank *P* value = 4.965e-13, **Figure 6A**), while the expression profile of these four genes within our classifier and survival status between two risk groups were visualized in **Figures 6D** **, E**. What’s more, the ROC curves for 5-year OS also revealed the same result that the risk score has the best predictive power of OS (AUC = 0.665) (**Figure 6B**). Besides, univariate and multivariate analysis still indicated that the classifier was significantly associated with OS after adjustment for clinical parameters in these validation sets (**Table 1**). Together, these findings suggested the CD8^+^ T cell-related classifier performed well in predicting the prognosis of stage III LUAD patients.

### The relationship between the classifier built with CD8^+^ T cell-related genes and clinicopathological parameters

**Figure 7:**
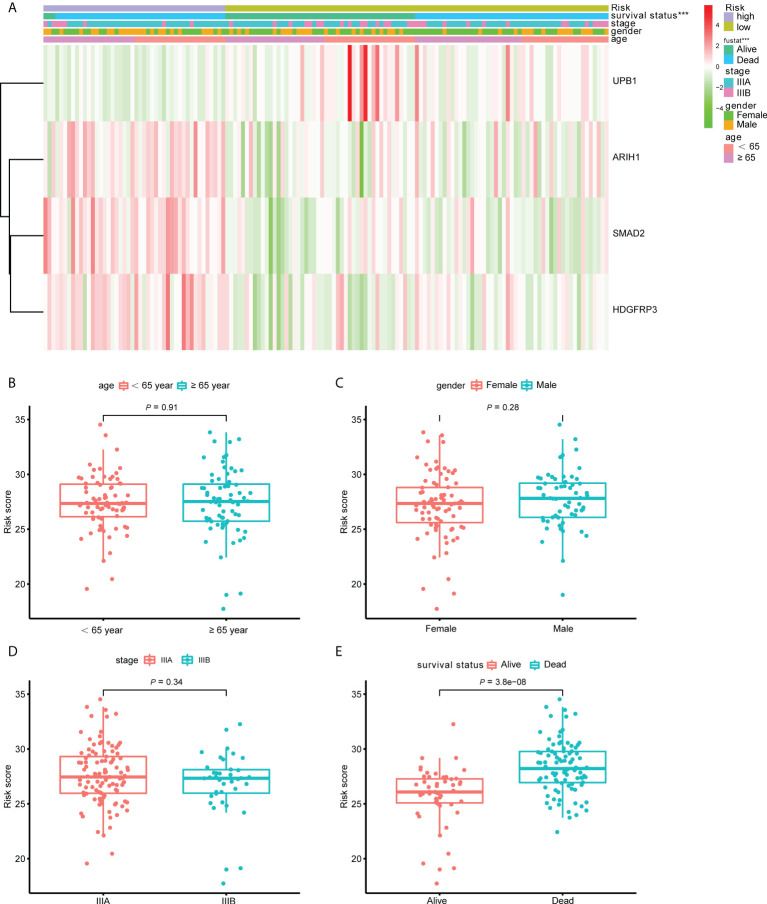
The correlation between the CD8^+^ T cell-related signature and clinical variables. **(A)** The heatmap revealed the association of the CD8^+^ T cell-related signature and the clinical variables (age, gender, stage and survival status) in the training set. **(B–E)** The box plots displayed the relationship between risk score and clinical features.

To better understand the clinical impact of the CD8^+^ T cell-related classifier in stage III LUAD patients, we analyzed the association of the signature with clinical variables in the training set. There was no significant association between the CD8^+^ T cell-related signature and TNM stage, gender, and age, apart from survival status (**Figure 7A**). What’s more, we further analyzed the comparison of risk scores in different subsets grouped by age, gender, TNM stage, and survival status. The risk scores were significantly different only in survival status subgroups, but not in age, stage, and gender subgroups (**Figures 7B** **–E**).

We next validate the prognostic ability of our CD8^+^ T cell-related classifier in different subsets clustered by clinicopathological variables. In the training set, patients with high-risk scores were inclined to have decreased survival rates, in the age, gender, and TNM stage subsets **(** **Supplementary Figures S5A-F**, *P* < 0.005**)**. Similar significant findings were revealed in the internal test set and the pooled test set, except in subsets of age less than 65-year and female in the internal test set **(** **Supplementary Figures S5G–L**, **S6**, *P* < 0.05**)**. As for the external test set, we also observed that the risk scores were significantly associated with unfavorable clinical outcomes in the age, gender, patients with positive node metastasis, T3+4, and TNM stage subsets **(** **Supplementary Figure S7**, *P* < 0.05**)**. These findings suggested that our CD8^+^ T cell-related classifier has a promising clinical application for selecting high-risk patients.

### Constructing a prognostic nomogram

**Figure 8:**
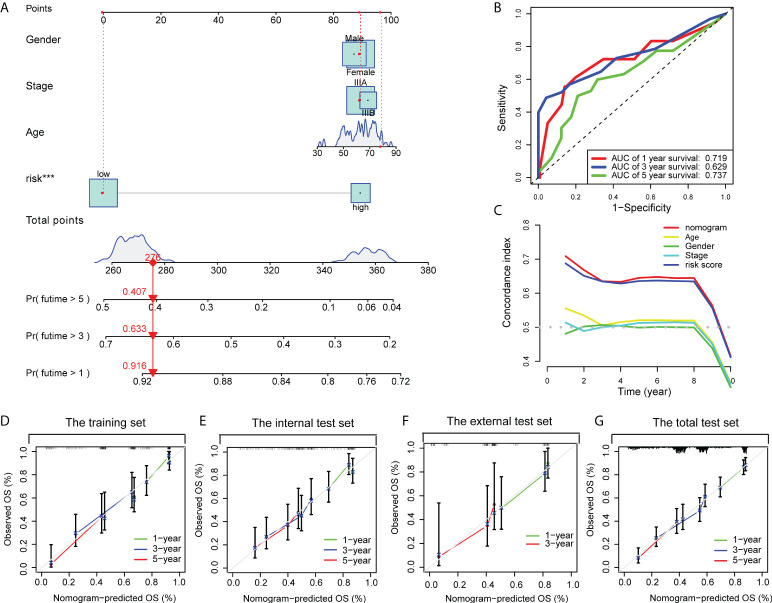
A nomogram was constructed to predict the OS. **(A)** A nomogram for predicting 1-, 3- and 5-year OS with risk levels and three clinical variables. **(B)** 1-, 3- and 5-year ROC curves of the nomogram for OS predictions. **(C)** The C index of the nomogram, risk signature and other clinical variables. Calibration plots of the nomogram for predicting probabilities of 1-year, 3-year, and 5-year OS of stage III LUAD patients in the training dataset **(D)**, the internal validation dataset **(E)**, the external validation dataset **(F)**, and the pooled validation dataset **(G)**.

By integrating the CD8^+^ T cell-related classifier and three clinicopathological features shared in the training dataset and the other validation datasets, we developed a prognostic nomogram to predict the 1-, 3-, and 5- year OS probability of LUAD patients in the training dataset **(** **Figure 8A** **)**. The AUC points of the nomogram for 1-, 3-, and 5-year survival predictions were 0.719, 0.629, and 0.737, respectively **(** **Figure 8B** **)**. The C-index indicated that the nomogram had the highest predictive accuracy of survival than the other clinicopathological parameters **(** **Figure 8C** **).** In addition, the calibration curves also confirmed a good consistency between predicted and observed scores in terms of probabilities of 1-, 3-, and 5-year OS **(** **Figure 8D** **)**. Similar results of calibration curves of nomogram were also found in the internal, external, and pooled validation datasets **(** **Figures 8E** **–G** **)**. Together, those findings indicated that our nomogram was suitable for clinical practice.

### Estimation of TIICs

**Figure 9:**
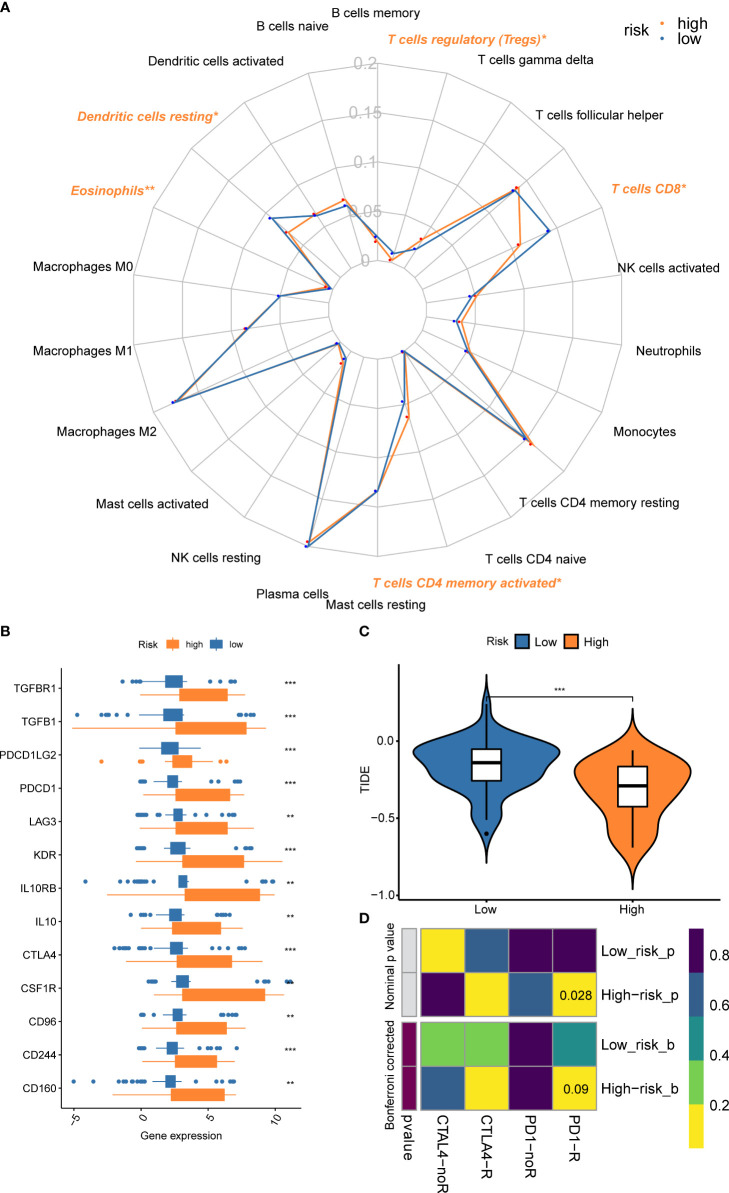
Comparison of the fractions of immune cells, expression of immune checkpoint genes, and immunotherapy benefits between risk subsets. **(A)** The radar plot revealed the 22 immune cell subpopulations between different risk groups; **(B)** The gene expression levels of immune checkpoint genes between risk groups; **(C)** Different benefits from the immunotherapy between risk subsets were predicted by the TIDE algorithm; **(D)** The submap algorithm indicated the probability of response to ICBs. **P < 0.01; ***P < 0.001.

Since CD8^+^ T cell-related classifier had closely and intrinsically connected with immune cells, which have a profound impact on predicting clinical outcomes and treatment efficacy, we further examined the difference and relationship of these immune cells with risk groups. The comparison of 22 immune cells between risk groups was displayed in a radar plot (**Figure 9A**). The results revealed that the abundance of CD8^+^ T cells was remarkably higher in the low-risk group compared with those in the high-risk group (*P* = 0.049). We also found the fractions of other immune cells, including Dendritic cells (DCs) resting, and Tregs were significantly increased in low-risk patients (*P* = 0.038, *P* = 0.028), whereas the expression levels of Eosinophils and T cells CD4 memory activated were obviously higher in the high-risk group (*P* = 0.004, *P* = 0.050) **(** **Supplementary Figures S8A** **–E** **)**.

### Prediction for efficacy of immunotherapy and chemo-agents

We also found the gene expressions of multiple immune checkpoint genes (ICGs), including CTLA-4, LAG3, PDCD1, CD96, CD244, and CSF1R, etc. were significantly increased in the high-risk group, which could be promising immunotherapy targets **(** **Figure 9B** **)**. To predict the response to immunotherapy, the TIDE algorithm was performed within different risk subsets. The result indicated that the TIDE score in the high-risk group was significantly lower than the low-risk group (**Figure 9C**), suggesting the patients within high-risk subsets could benefit from the immunotherapy. A similar result was observed in the submap algorithm. The high-risk subset showed a higher probability of response to PD-1 blockades (Nominal *P* = 0.028) (**Figure 9D**).

Chemotherapy is another common therapy for stage III LUAD, while a higher IC50 value indicates resistance to the drug, otherwise, it is sensitive to the drug. The results showed that the IC50 values of cisplatin and gemcitabine decreased significantly in the high-risk subset; The IC50 values of docetaxel and paclitaxel had a decreased trend in the high-risk subset, although there was no significant difference (**Supplementary Figures S9A** **–D**); Overall, these findings suggested that the stage III LUAD patients from the high-risk subset would benefit from immunotherapy and chemotherapy.

### Validation of the gene expression including in the classifier and evaluating the efficacy of immunotherapy in a real-world cohort

**Figure 10:**
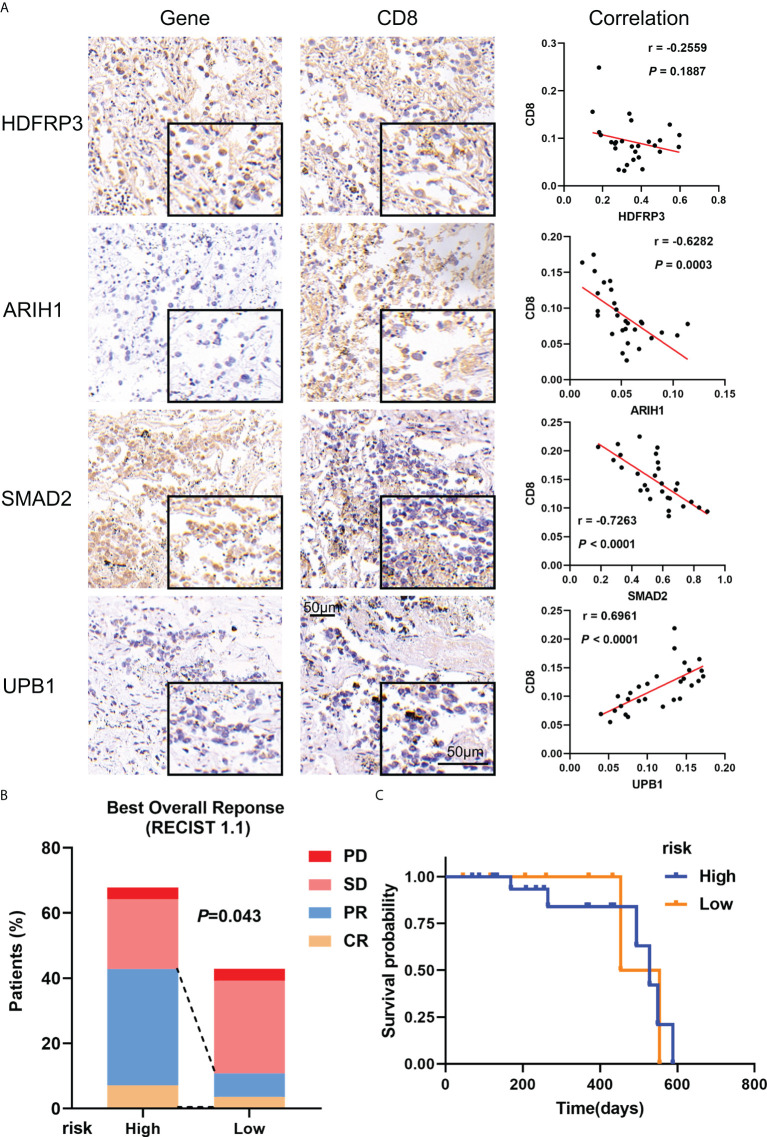
Verification of gene expression including in the model and its validity in real-world cohorts. **(A)** Representative microphotographs of gene staining included in the model and the correlation between these genes and CD8 in stage III LUAD. **(B)** The recent therapeutic effect of immunotherapy between risk groups; **(C)** KM curves for OS between risk groups.

To verify the consistency of the model across cohorts and its validity for clinical application, a real-world cohort was constructed. 28 patients were enrolled in the cohorts, immunohistochemical detection showed that the expression of ARIH, and SMAD2 had a negative correlation with CD8 in LUAD tissues (r = -0.6282, *P* = 0.0003; r = -0.7263, *P* < 0.0001), while the expression of UPB1 had a positive correlation with CD8 (r = 0.6961, *P* < 0.0001), which were consistent with the results obtained based on open databases (**Figure 10A**, **Supplementary Figure S10**). For the expression of HDFRP3, unfortunately, we only got a negative trend with CD8 (r = -0.2559), but without significant statistical significance, probably due to the small sample size. To further validate whether the model could well predict the efficacy of immunotherapy for stage III LUAD patients in the real world, we calculated the risk score based on the gene-positive staining IOD/Area obtained in immunohistochemistry analysis and subsequently divided these patients into high- and low-risk groups. After the Chi-square test, all the clinicopathological factors were well balanced between the two groups (*P* > 0.05) (**Supplementary Table S2**). Furthermore, we evaluated the effectiveness of immunotherapy for each patient objectively. In the high-risk group, 2 of 19 patients reached CR during treatment, 10 patients achieved PR, 6 patients reached SD, and 1 patient reached PD, while 1 of 9 patients reached CR, 1 patient achieved PR, 6 patients achieved SD, and 1 patient achieved PD in the low-risk groups (**Figure 10B**). The overall response rate (ORR) was significantly higher in the high-risk group (63.16% vs 22.22%, *P* = 0.043) (**Supplementary Table S3**). However, probably due to the short follow-up time and the small number of cases, we did not observe a survival difference between the two groups (**Figure 10C**). Overall, we confirmed the validity of the model in a real-world cohort and its clinical applicability.

## Discussion

The heterogeneity of stage III LUAD is not only reflected in the wide range of tumor size (T_1_-T_4_), the degree of local tumor invasion, and the involvement of ipsilateral or contralateral mediastinal lymph nodes (N_0_-N_3_) (3), but also in the diverse tumor molecular mutations in the histopathological type of LUAD. Thus, the 5-year survival rates are generally poor and have a varying range from 12% to 36% in the pathological stage (30), however, some clinical trials still cannot explain the exact reasons (31). Consequently, just the commonly used prognostic indicators, such as tumor stage and patient’s general condition, are not well appropriate for this group of patients. Besides, the ICBs such as durvalumab have shown good survival benefits in stage III NSCLC (8), but due to the obvious heterogeneity in immune features (32), the PD-L1 expression, as well as TMB, are not good predictors of immunotherapy efficacy, which is different from their application in stage IV of NSCLC (7). Because of significant role of CD8^+^ T cell in anti-cancer immunotherapy, hence, finding new biomarkers and constructing a CD8^+^ T cell-related classifier to predict the prognosis and effect of immunotherapy have a significant meaning for patients with stage III LUAD.

Therefore, in this study, we extracted all RNA level profiles of these locally advanced LUAD patients from the GEO, Array Express, and TCGA database and divided these patients into the training, internal validation, external validation, and pooled validation cohorts firstly. Then we constructed the gene co-expression network through identified a significant yellow module as a hub module that exhibited great relevance to CD8^+^ T cells by CIBERSORT and WGCNA analysis. Next, through univariate Cox analysis, lasso analysis, and multivariate Cox analysis, a CD8^+^ T cell-related signature including HDFRP3, ARIH1, SMAD2, and UPB1 was constructed. It was gratifying that this model could divide locally advanced LUAD patients into low- and high-risk groups with distinct overall survival in multiple cohorts (all *P* < 0.05). What’s more, in comparisons of the age, gender, and stage, the area under the ROC curve of the model was always the largest. Moreover, to make this model better applicable in the clinic, a nomogram including the traditional clinical parameters and risk signature was constructed. The ROC, C-index, and calibration curves validated its robust predictive capacity very well. Meanwhile, KM analysis revealed a significant difference in the subgroup analyses’ survival between the two risk subsets, especially in different TNM stages, suggesting the robust clinical application of our CD8^+^ T cell-related classifier. Finally, we confirmed that the high-risk group might benefit from immunotherapy or chemotherapy, and verified the valuation of this model in a real-world cohort, which further clarified the value of the model in predicting efficacy.

Specifically, as the protective factor included in this model, the most important function of UPBEAT1 (UPB1) is that it could directly regulate the expression of a set of peroxidases which modulates the balance of reactive oxygen species (ROS) (33). Besides, for cancer patients, UPB1 was screened as a prognostic circulating biomarker or signature for patients with hepatocellular carcinoma (34, 35), similar to clear renal cell carcinoma (36). In addition, for the treatment of specific tumors, especially the 5-fluorouracil treatment of colorectal cancer, some researchers explored the role of UPB1 in the 5-fluorouracil pathway or fluoropyrimidine-related high toxicity (37, 38). What’s more, we not only introduced UPB1 in the clinical prognostic analysis of LUAD for the first time but also found the expression of UPB1 was correlated with CD8^+^ T cells. In the follow-up mechanism exploration, whether UPB1 affects CD8^+^ T cells in LUAD by regulating the expression of ROS is a direction worth studying.

In contrast to the protective factor UPB1, we included three risk genes in the model, namely SMAD2, ARIH1, and HDGFRP3. Among them, the most important and valuable biomarker was SMAD2. As a transcription factor member of the SMADs family, SMAD2 is activated by receptors such as TGF-β mediated phosphorylation, which plays a critical role in transmitting the TGF-β superfamily from the cell surface to the nucleus in turn (39). TGF-β/SMAD signaling is considered to culminate in the suppression of tumor-specific cellular immunity, which performs functions in a variety of cells. For CD8^+^ T cell, Gunderson et al. found that TGF-β increased the binding of Smad2 and reduced CXCR3 expression in CD8^+^ T cells, thereby limiting their trafficking into tumors (40). Li et al. reported that Icaritin reduced CD8^+^ T cell chemotaxis by inhibiting the CXCL10/CXCR3 axis and suppressing the TGF-β/Smad2 signaling pathway in COPD (41). Furthermore, Park et al. found that TGF-β1 mediated SMAD3 to enhance PD-1 expression on antigen-specific T cells resulting in T cell suppression (42). However, the specific mechanism by which SMAD2 affects CD8^+^ T cells in LUAD remains unclear. Besides, SMAD2 phosphorylation was observed after activation in the Treg, which could produce the bioactive form of TGF-β (43). For cancer cells, Vimentin consequently led to metastasis and immune escape through the expression of PD-L1 in LUAD by triggering the TGF-β/SMAD2 signaling (44). In addition, ARIH1 (or HHARI) known as a ubiquitin-protein ligase, contributed to EMT induction and breast cancer progression (45, 46). However, Wu et al. found that the overexpression of ARIH1 could suppress tumor growth and promote cytotoxic T cell activation by inducing PD-L1 degradation (47). Also, a few reports indicated that high expression of HDGFRP3 (or HRP-3) promoted hepatocellular carcinomas and identified it was associated with metastasis in breast cancer (48, 49). In summary, the four genes included in the model have not yet been reported to be associated with CD8^+^ T cells in LUAD, which means that these biomarkers are important innovations for antitumor immunotherapy research.

Moreover, the results of GO and KEGG enrichment analysis demonstrated a meaningful finding that these genes picked from the hub yellow module were critically enriched in ubiquitin protein ligase binding, SMAD binding, and lymphocyte activation. It was gratifying that an E3 ubiquitin ligase such as ARIH1 could promote anti-tumor immunity *via* PD-L1 degradation, thereby affecting T cell activation, which has been mentioned above (47). This finding also strongly supports the value of such a CD8^+^ T cell-based predictive model proposed in this study in predicting the efficacy of immunotherapy. As for the SMAD2 normally coupled with SMAD3 mentioned above, its functions were mainly embodied through the key signaling axis containing transcription factor Forkhead box protein P1 (Foxp1) and TGF-β in the tumor immune microenvironment. For instance, Foxp1 interacted with Smad2/3 and suppressed the tumor-reactive T cells’ response to TGF-β in advanced tumors (50). Hence, the regulation of TGF-β/SMAD signaling function is important for developing new immunotherapeutic strategies by restoring the immunosuppressive TME to active status (51). For example, using the genetic method to modify antigen-specific T cells by interfering with TGF-β signaling would significantly enhance tumor treatment efficacy (52). Mesenchymal stem cells secreted TGF-β induced the differentiation of Treg cells *via* SMAD2 as so to inhibit colorectal cancer (53). A similar function was also reflected in the inhibition of IL-2 which was regarded as a key cytokine for T cell proliferation and activation (54). These findings also support the potential application of the model in assessing the prognosis of immunotherapy. Furthermore, it is worth further exploring whether the other genes involved in the model are associated with the phenotype of lymphocytes, especially CD8^+^ T cells.

The critical role of TME is beyond doubt in tumor initiation and development. Although studies have facilitated the identification of the important functions of different immune cell subtypes within TME, the CD8^+^ T cell is the central focus in engaging adaptive immunity for cancer control according to the cancer-immunity cycle (14, 55). Moreover, the number and functionality of CD8^+^ T cells after activation are prerequisites for the efficacy of immunotherapy in patients with lung cancer (56). Given our classifier constructed from CD8^+^ T cell-related genes, the risk score of the CD8^+^ T cell-related classifier was consistent with the expectation and negatively related to the abundance of CD8^+^ T cells, while patients within the high-risk group were associated with poor survival status. Besides, we also found other subclasses, such as resting DCs, and regulatory T cells (Tregs), were obviously decreased in the high-risk group. Owing to the unique capacity in initiation and regulation of T cell responses, DCs have been extensively explored as tools for immunotherapy, therefore it was convinced that a decrease in DCs is associated with poor prognosis (57, 58). Nevertheless, high-risk patients possessed a higher fraction of activated memory CD4^+^ T cells and eosinophils. Particularly, the count and percentage of eosinophils significantly increased in NSCLC patients treated with ICBs, and metastasis-entrained eosinophils could enhance lymphocyte-mediated antitumor immunity, which might somehow explain the reason why high-risk group patients could benefit from immunotherapy (59, 60). Besides, the higher expression of immune checkpoint genes in the high-risk group also could indicate the benefits of immunotherapy (61). Overall, such a risk score of CD8^+^ T cell-related classifier was significantly correlated with multiple immune cell subtypes, which provided important hints for revealing the interaction between immune cells and tumor cells in the TME, as well as between different immune cell subtypes.

In recent years, based on the fact that immunotherapy has benefited some patients with locally advanced or advanced NSCLC and with the rapid development of bioinformatics, more researchers turned their attention to discovering some models that integrated multi-factors to better predict the survival rates and evaluate the benefits of immunotherapy. Several studies have proposed immune prognostic models involving multiple genes that could evaluate the prognosis of patients with LUAD, however, they did not specify which types of immune cells these genes were associated with, nor did they separately analyze the patients with locally advanced LUAD (62–64). Besides, Xie et al. developed a nomogram for LUAD patients based on immune scores and concluded high score was related to better OS, but immunotherapy was not involved and external data verification was needed (65). Zhang et al. established a CD8^+^ T cell-associated gene signature, which could help assess prognostic risk and immunotherapy response in LUAD patients. However, they did not validate the signature with real-world samples (66). Moreover, some researchers demonstrated the tumor immune microenvironment by analyzing the targeted RNA-Seq of immune-related genes, which had prognostic value for locally advanced LUAD (67). Unfortunately, the effective value was limited by the small sample size from a single institution. Thus, these models have a few limitations and insufficient predictive power for locally advanced LUAD.

Although we have constructed a risk model and a nomogram based on this, which has good predictive efficacy in survival rates and potential application in the prediction of immunotherapy or chemotherapy efficacy in locally advanced LUAD. Nevertheless, there are also some limitations of this study. First, to incorporate more data into our research, we have selected as many data sets as possible in the GEO database, although they contained several different platforms. Thus, such a fusion of multiple data might increase the possibility of over-correction in the data processing. In addition, we have only repeatedly verified the effectiveness of the model through different open cohorts. Although we validated the model in a real-world cohort, we did not obtain particularly significant differences due to, for example, the small number of cases. Overall, further experimental validations are needed to determine whether these genes included in the model are involved in the progression of locally advanced LUAD and how they affect the phenotypes of CD8^+^ T cells.

In summary, based on the multiple cohorts, we have constructed a prediction model correlated to CD8^+^ T cell and the nomogram in patients with locally advanced LUAD. Furthermore, the overwhelming impression of our study was the better effectiveness and accuracy of the model in predicting survival rates and immunotherapy efficacy by designing multiple validation cohorts from open or real-world databases. Hence, based on CD8^+^ T cell-related genes in the model, if the mechanism of the relationship between the level of risk factors and the CD8^+^ T cell phenotypes could be explored, then such a model will be better applied to predict the prognosis of locally advanced LUAD patients on immunotherapy and enable patients to benefit from treatments.

## Data availability statement

The original contributions presented in the study are included in the article/**Supplementary Material**. Further inquiries can be directed to the corresponding author.

## Ethics statement

The studies involving human participants were reviewed and approved by the Research Ethics Committees of the First Affiliated Hospital of Xi’an Jiaotong University. The patients/participants provided their written informed consent to participate in this study.

## Author contributions

(I) Conception and design: JF, HG. (II) Administrative support: HG. (III) Provision of study materials: JF, LX, SZ. (IV) Collection and assembly of data: JF, SZ. (V) Data analysis and interpretation: JF, LX. (VI) Experimental validation: JF, TZ, YY. (VII) Manuscript writing: All authors. (VIII) Final approval of manuscript: All authors.

## Funding

This work was supported by Interdisciplinary Training Program for Doctoral Candidate of Xi’an Jiaotong University (IDT1919), Guangdong Association of Clinical Trials (GACT)/Chinese Thoracic Oncology Group (CTONG) and Guangdong Provincial Key Lab of Translational Medicine in Lung Cancer (Grant No.2017B030314120), Natural Science Foundation of Shaanxi Province (No.2019JM-559), and Xi’an Jiaotong University Free Exploration and Innovation Program (Student Category) (No.sxzy022021011).

## Acknowledgments

All authors would like to express our sincere thanks for sharing the online databases.

## Conflict of interest

The authors declare that the research was conducted in the absence of any commercial or financial relationships that could be construed as a potential conflict of interest.

## Publisher’s note

All claims expressed in this article are solely those of the authors and do not necessarily represent those of their affiliated organizations, or those of the publisher, the editors and the reviewers. Any product that may be evaluated in this article, or claim that may be made by its manufacturer, is not guaranteed or endorsed by the publisher.

## Supplementary material

Supplementary Figure 1 The selection process for inclusion of patients as the real-world cohort.

Supplementary Figure 2 The before and after batch corrections were displayed by PCA plots.

Supplementary Figure 3 WGCNA for construction and validation of the hub module. **(A)** Scale-free fit with the soft threshold power from 1-20 (x-axis) and the corresponding signed R^2^ (y-axis); **(B)** Mean connectivity analysis for 1-20 soft threshold power; **(C)** CD8^+^ T cell-related genes were grouped into different modules marked with various colors *via* hierarchical clustering tree.

Supplementary Figure 4 The feature notes of hub modules. **(A)** The heatmap exhibited the correlations of modules with T cells infiltration; **(B)** The top 20 enriched terms were shown as a bar chart; **(C)** The network was constructed for these enriched terms.

Supplementary Figure 5 KM curves for OS indicated prognostic power of the CD8^+^ T cell-related signature in various subsets of the training cohort and the internal test set. **(A)** age < 65; **(B)** age ≥ 65; **(C)** Female; **(D)** Male; **(E)** stage IIIA; **(F)** stage IIIB; **(G–L)** for the internal test set was similar to (A-F) for the training cohort.

Supplementary Figure 6 KM curves for OS indicated prognostic power of the CD8^+^ T cell-related signature in the pooled test set. **(A)** age < 65; **(B)** age ≥ 65; **(C)** Female; **(D)** Male; **(E)** stage IIIA; **(F)** stage IIIB.

Supplementary Figure 7 KM curves for OS indicated prognostic power of the CD8^+^ T cell-related signature in the external test set. **(A)** age < 65; **(B)** age ≥ 65; **(C)** Female; **(D)** Male; **(E)** Negative nodes; **(F)** Positive nodes; **(G)** stage T1+T2; **(H)** stage T3+T4; **(I)** stage IIIA; **(J)** stage IIIB.

Supplementary Figure 8 Quantitative differences of immune cell subtypes between risk groups. **(A)** Resting dendritic cells; **(B)** CD8^+^ T cells; **(C)** Tregs; **(D)** Eosinophils; **(E)** Activated memory CD4^+^ T cells.

Supplementary Figure 9 Chemotherapy benefits stratified by different risk subsets. **(A–D)** IC50 plots of chemo-agents between the two subsets. **(A)** Cisplatin; **(B)** Docetaxel; **(C)** Gemcitabine; **(D)** Paclitaxel.

Supplementary Figure 10 Correlation of genes included in the model with the Tumor-Infiltrating Immune Cells.

## Abbreviations

NSCLC, non–small-cell lung cancer; LUAD, lung adenocarcinoma; OS, overall survival; PD-1, programmed death 1; PD-L1, programmed death-ligand 1; ICBs, immune checkpoint blockades; TME, tumor microenvironment; WGCNA, weighted correlation network analysis; GEO, Gene Expression Omnibus; TCGA, The Cancer Genome Atlas; KM, Kaplan-Meier; ROC, receiver operating characteristic; C-index, concordance index; t-ROC, time-dependent ROC; TIDE, tumor immune dysfunction and exclusion; TIICs, tumor-infiltrating immune cells; DCs, dendritic cells; Tregs, regulatory T cells; BOR, best overall response; ICGs, immune checkpoint genes; ORR, overall response rate; CR, complete response; PR, partial response; SD, stable disease; PD, progressive disease.
