# Supplementary figures and images for: A robust CD8+ T cell-related classifier for predicting the prognosis and efficacy of immunotherapy in stage III lung adenocarcinoma
Source: Front Immunol. 2022 Aug 31;13:993187. doi: 10.3389/fimmu.2022.993187 (PMC9471021; doi:10.3389/fimmu.2022.993187)

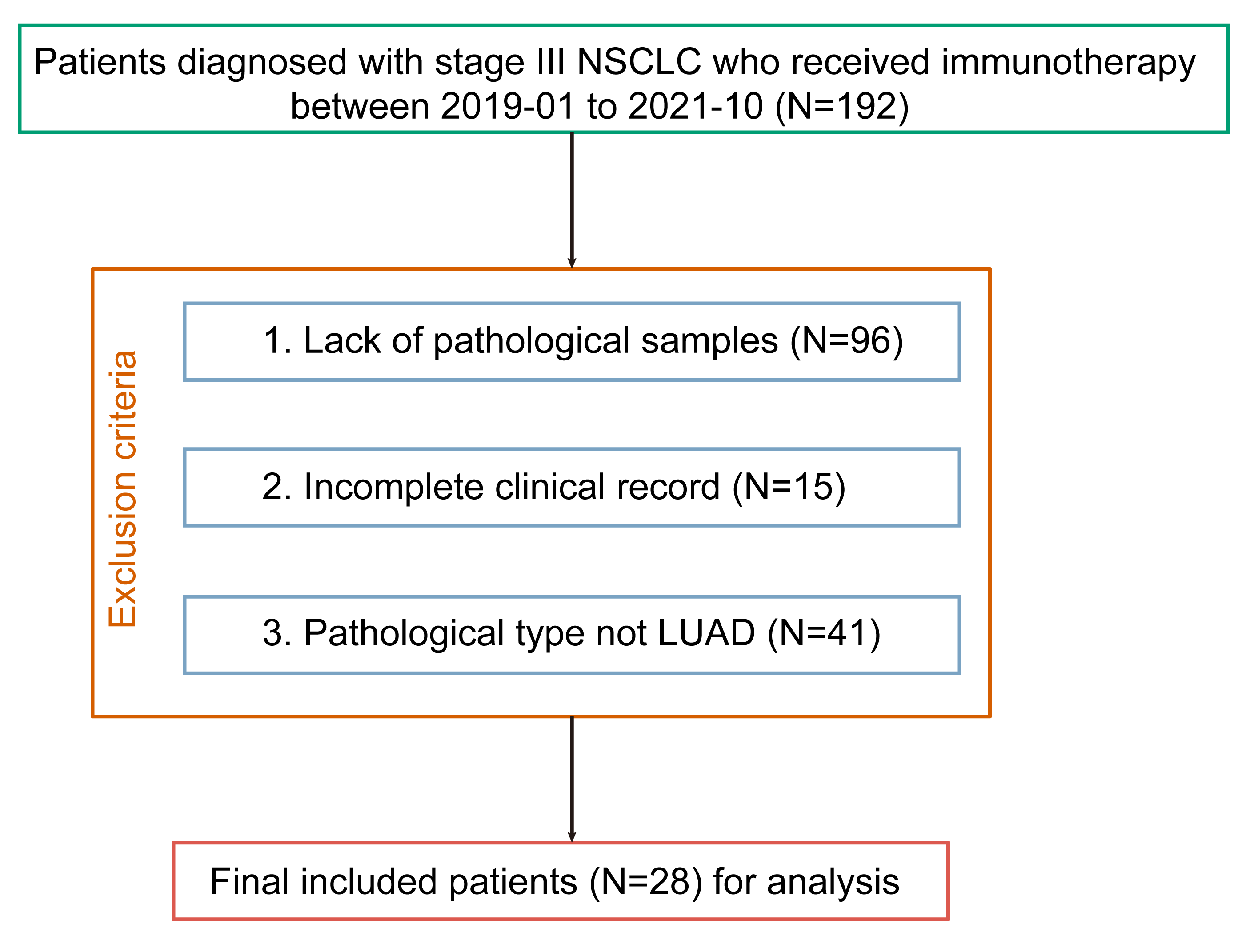

Supplement: Supplementary Figure 1 — The selection process for inclusion of patients as the real-world cohort. [file Image_1.tif]

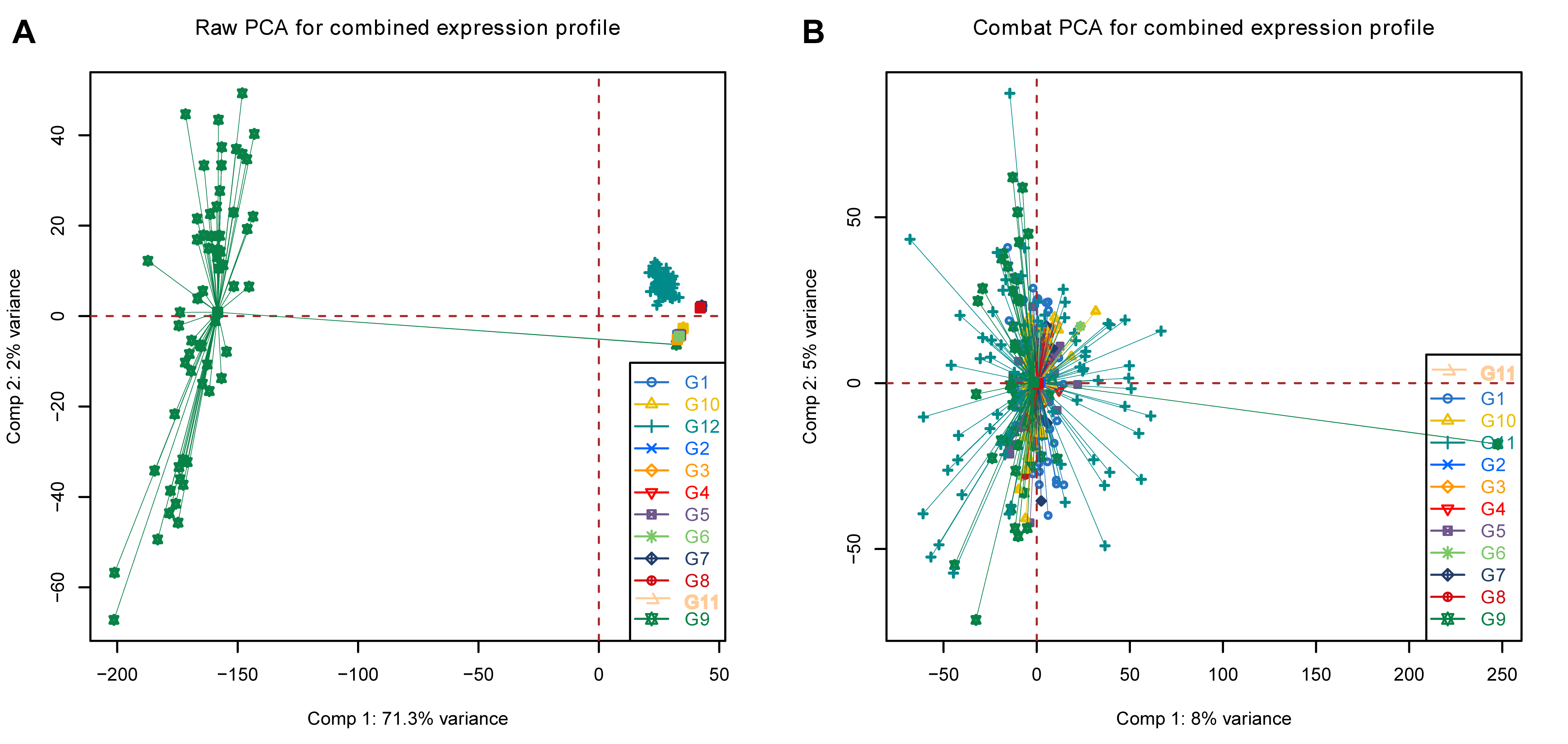

Supplement: Supplementary Figure 2 — The before and after batch corrections were displayed by PCA plots. [file Image_2.tif]

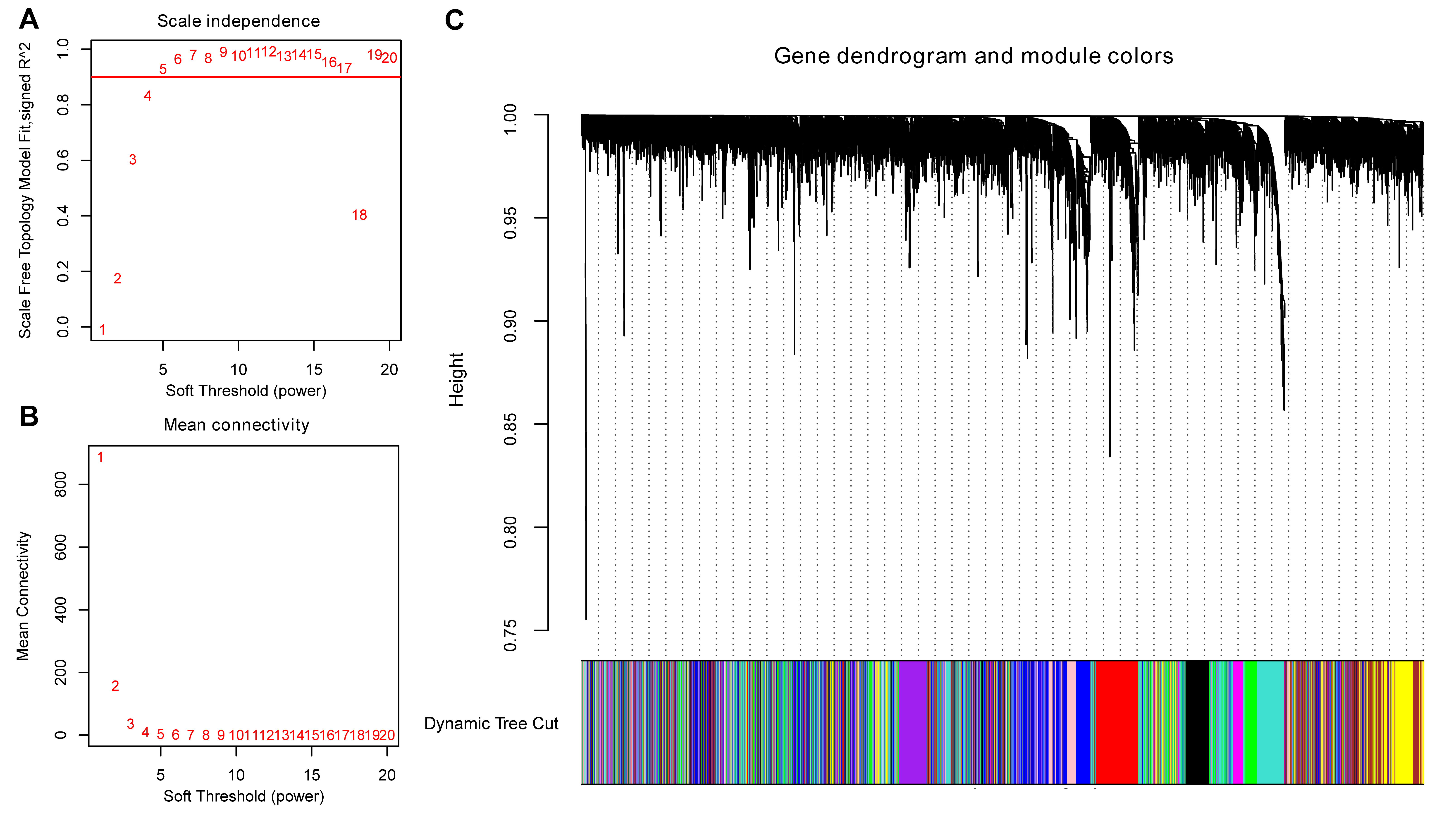

Supplement: Supplementary Figure 3 — WGCNA for construction and validation of the hub module. (A) Scale-free fit with the soft threshold power from 1-20 (x-axis) and the corresponding signed R2 (y-axis); (B) Mean connectivity analysis for 1-20 soft threshold power; (C) CD8+ T cell-related genes were grouped into different modules marked with various colors via hierarchical clustering tree. [file Image_3.tif]

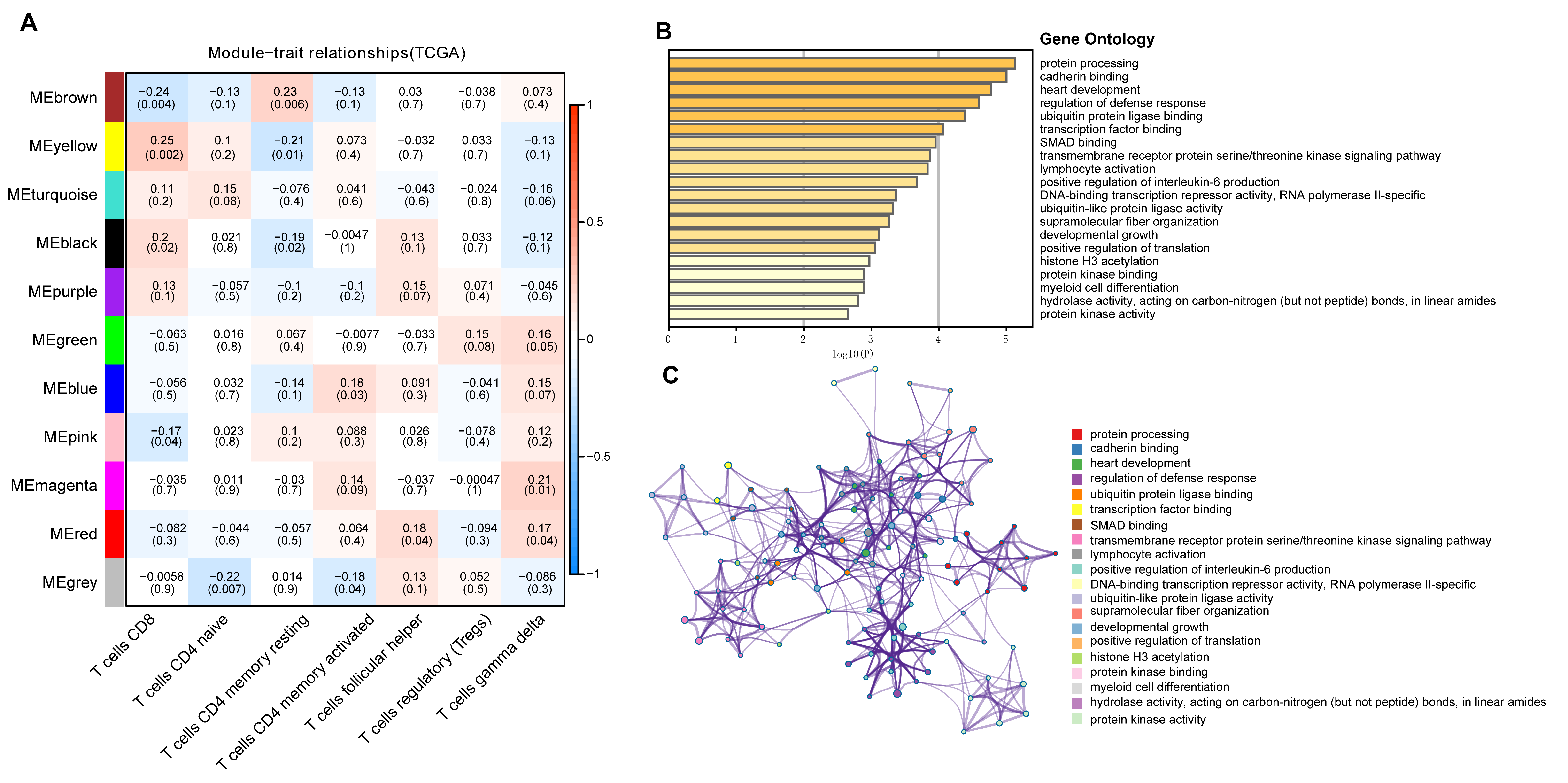

Supplement: Supplementary Figure 4 — The feature notes of hub modules. (A) The heatmap exhibited the correlations of modules with T cells infiltration; (B) The top 20 enriched terms were shown as a bar chart; (C) The network was constructed for these enriched terms. [file Image_4.tif]

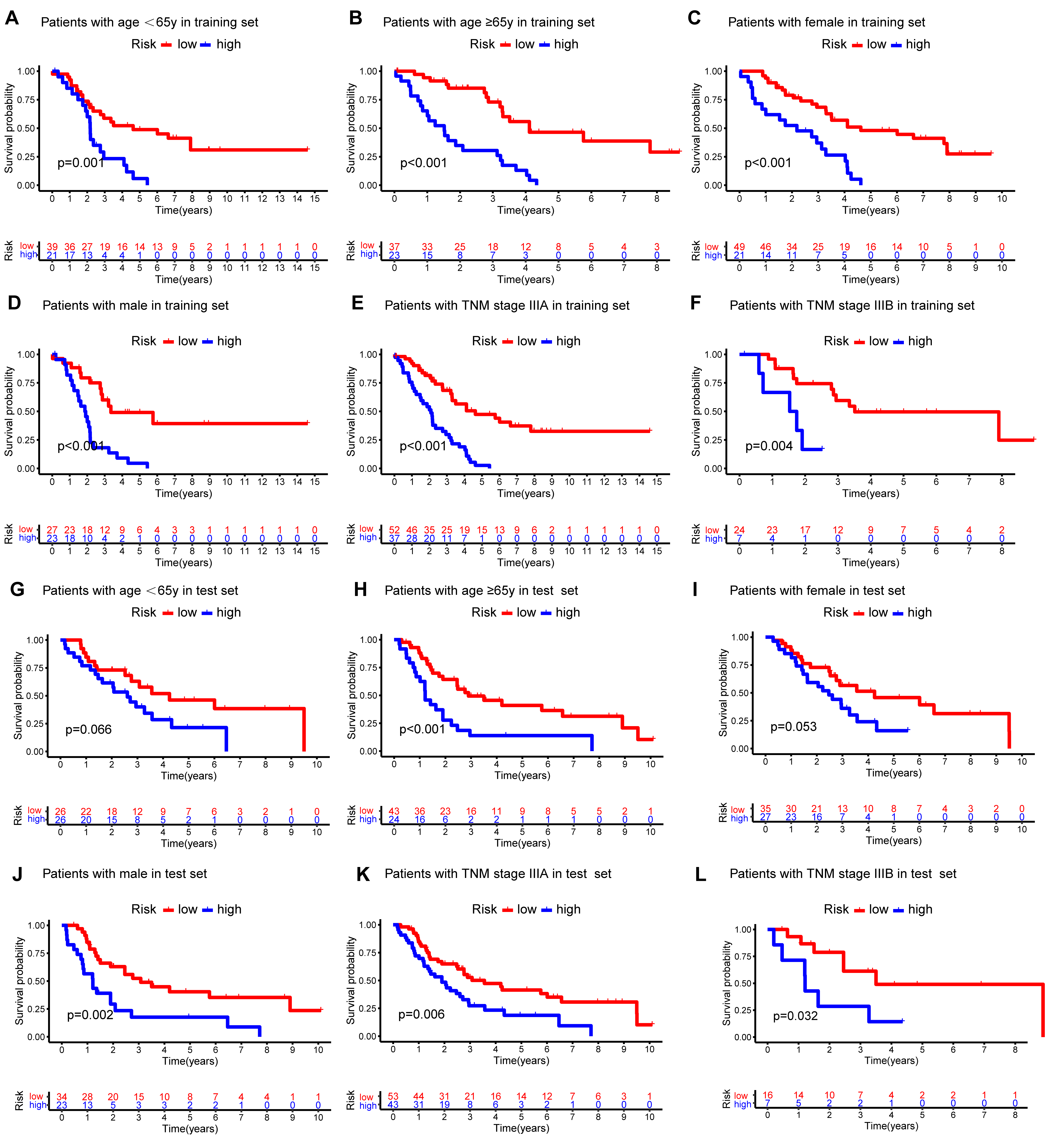

Supplement: Supplementary Figure 5 — KM curves for OS indicated prognostic power of the CD8+ T cell-related signature in various subsets of the training cohort and the internal test set. (A) age < 65; (B) age ≥ 65; (C) Female; (D) Male; (E) stage IIIA; (F) stage IIIB; (G–L) for the internal test set was similar to (A-F) for the training cohort. [file Image_5.tif]

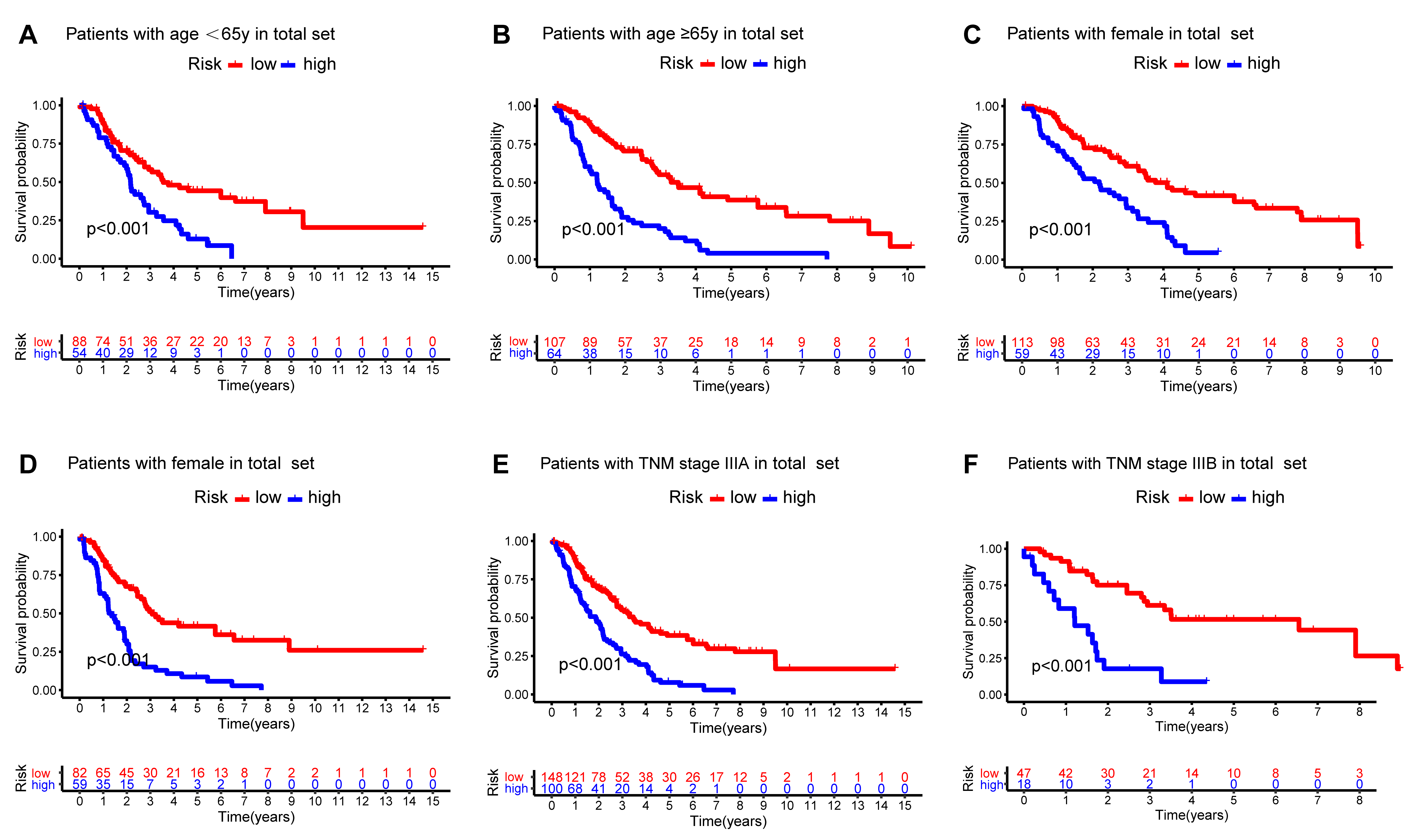

Supplement: Supplementary Figure 6 — KM curves for OS indicated prognostic power of the CD8+ T cell-related signature in the pooled test set. (A) age < 65; (B) age ≥ 65; (C) Female; (D) Male; (E) stage IIIA; (F) stage IIIB. [file Image_6.tif]

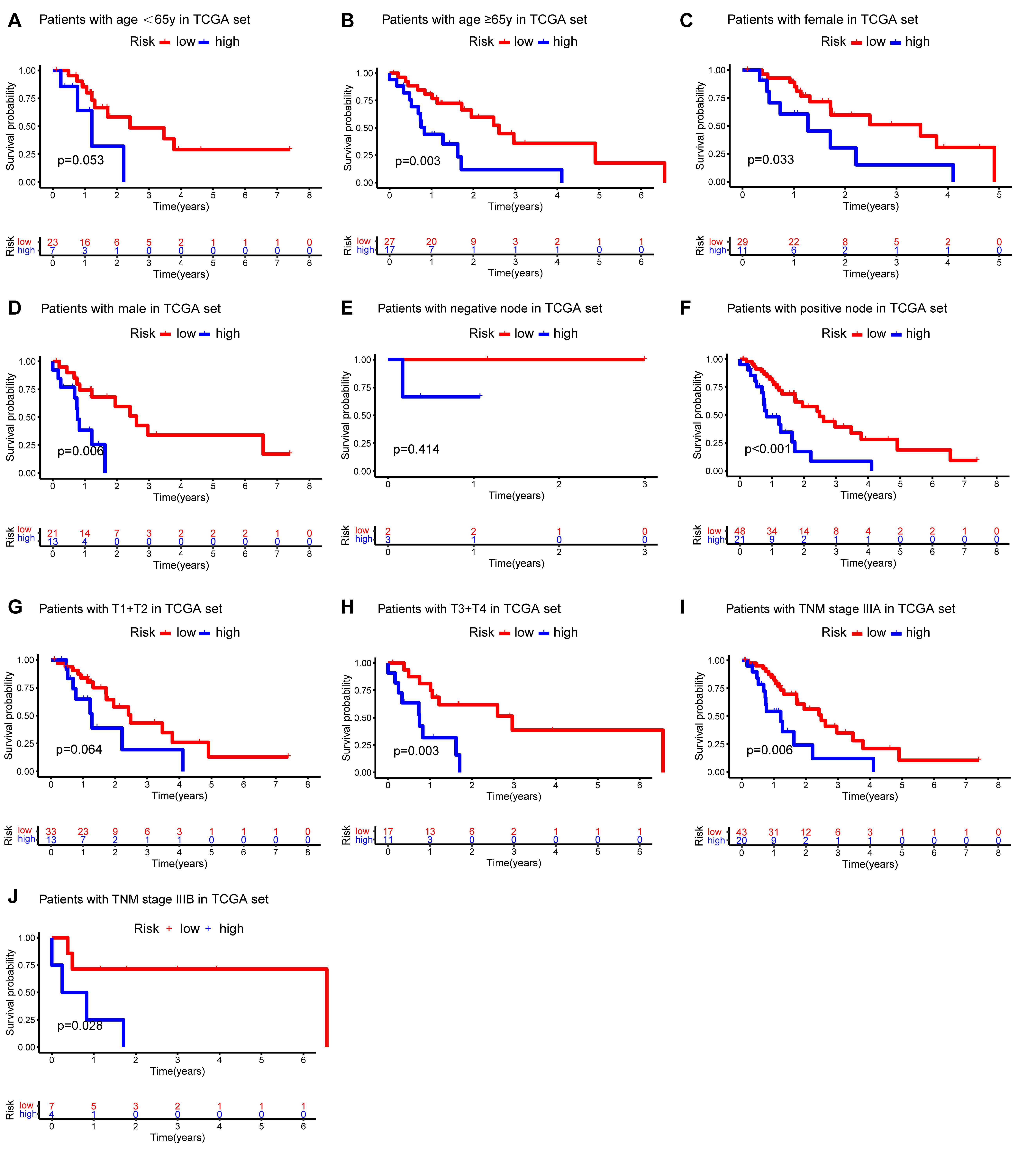

Supplement: Supplementary Figure 7 — KM curves for OS indicated prognostic power of the CD8+ T cell-related signature in the external test set. (A) age < 65; (B) age ≥ 65; (C) Female; (D) Male; (E) Negative nodes; (F) Positive nodes; (G) stage T1+T2; (H) stage T3+T4; (I) stage IIIA; (J) stage IIIB. [file Image_7.tif]

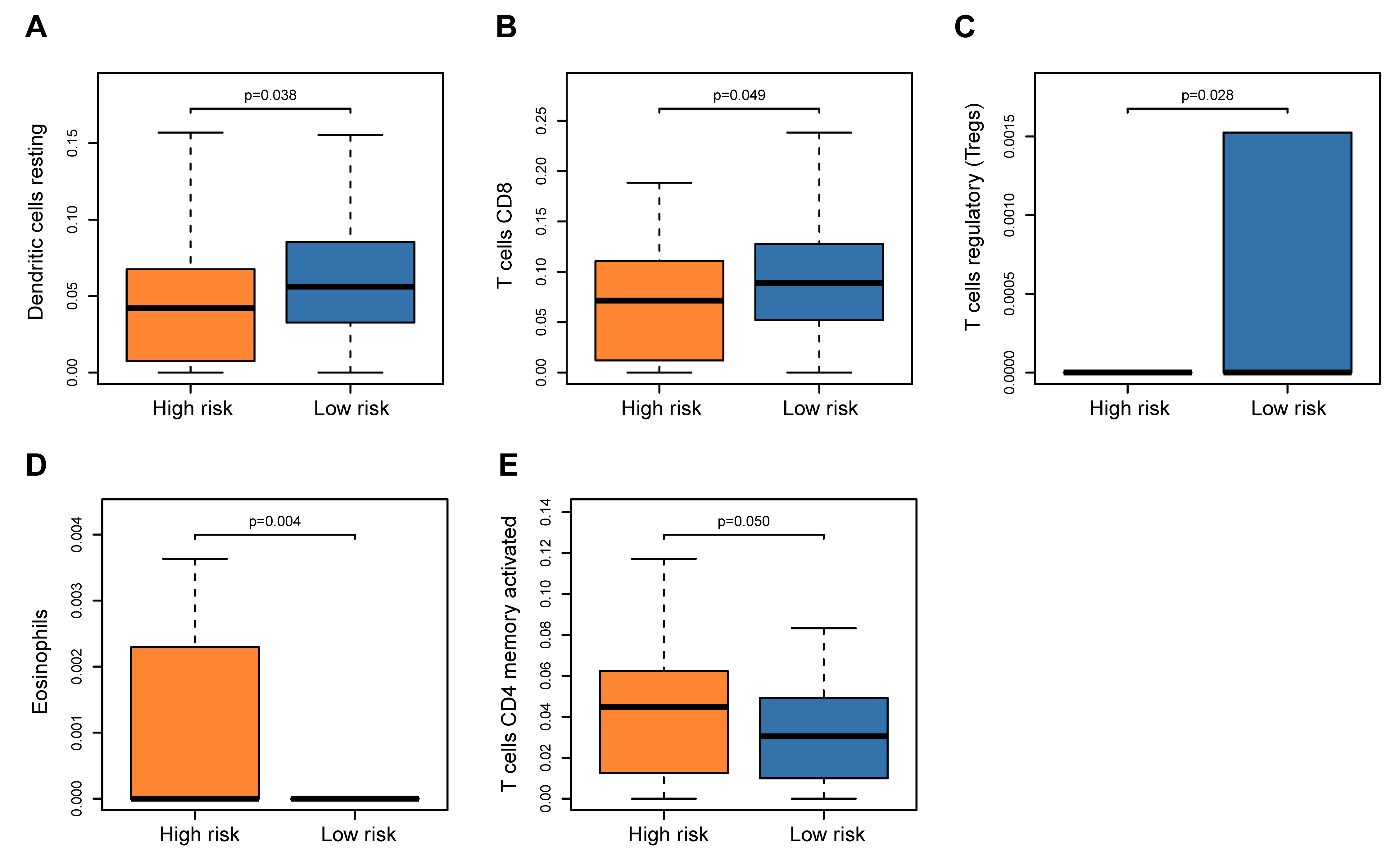

Supplement: Supplementary Figure 8 — Quantitative differences of immune cell subtypes between risk groups. (A) Resting dendritic cells; (B) CD8+ T cells; (C) Tregs; (D) Eosinophils; (E) Activated memory CD4+ T cells. [file Image_8.tif]

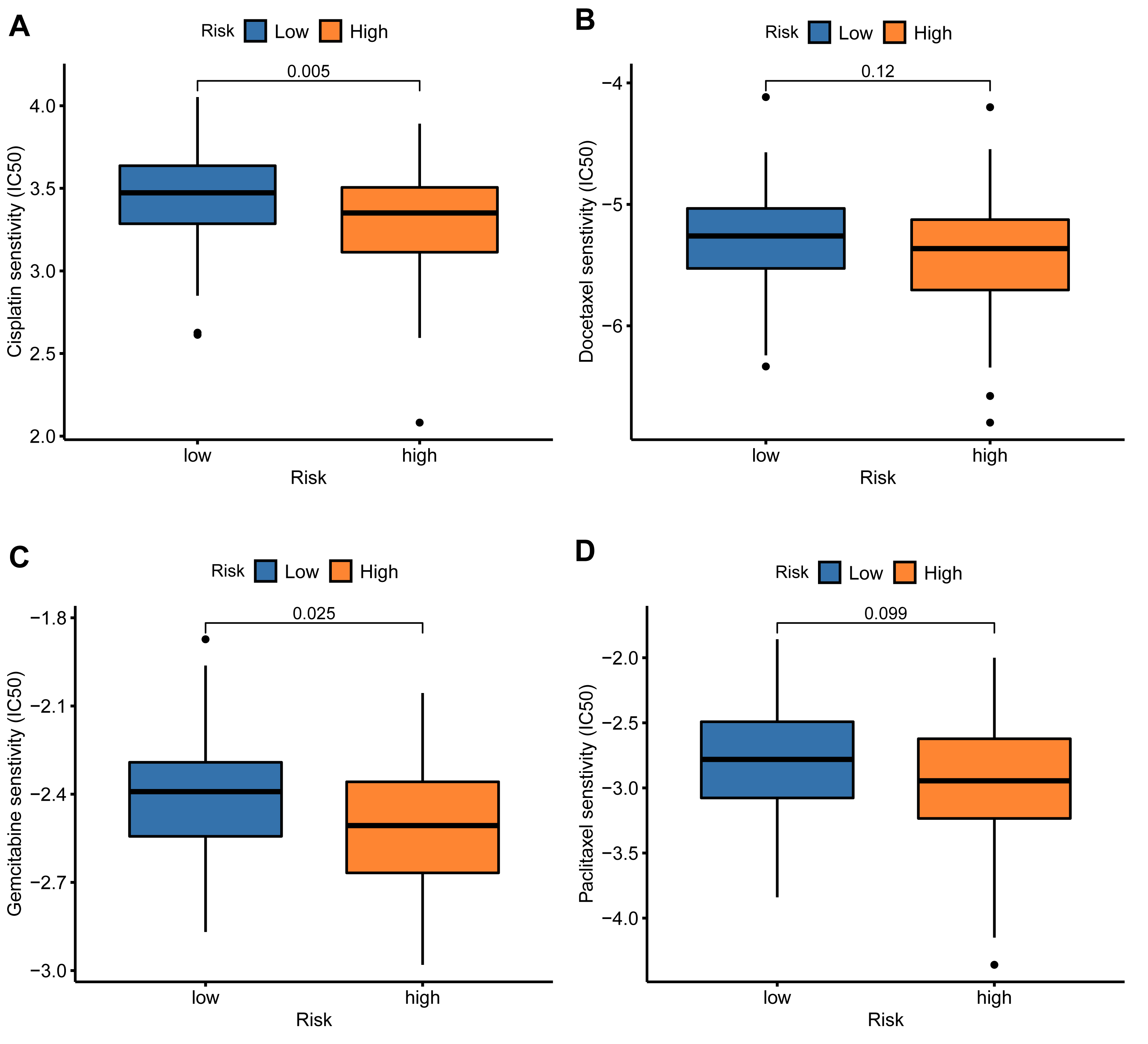

Supplement: Supplementary Figure 9 — Chemotherapy benefits stratified by different risk subsets. (A–D) IC50 plots of chemo-agents between the two subsets. (A) Cisplatin; (B) Docetaxel; (C) Gemcitabine; (D) Paclitaxel. [file Image_9.tif]

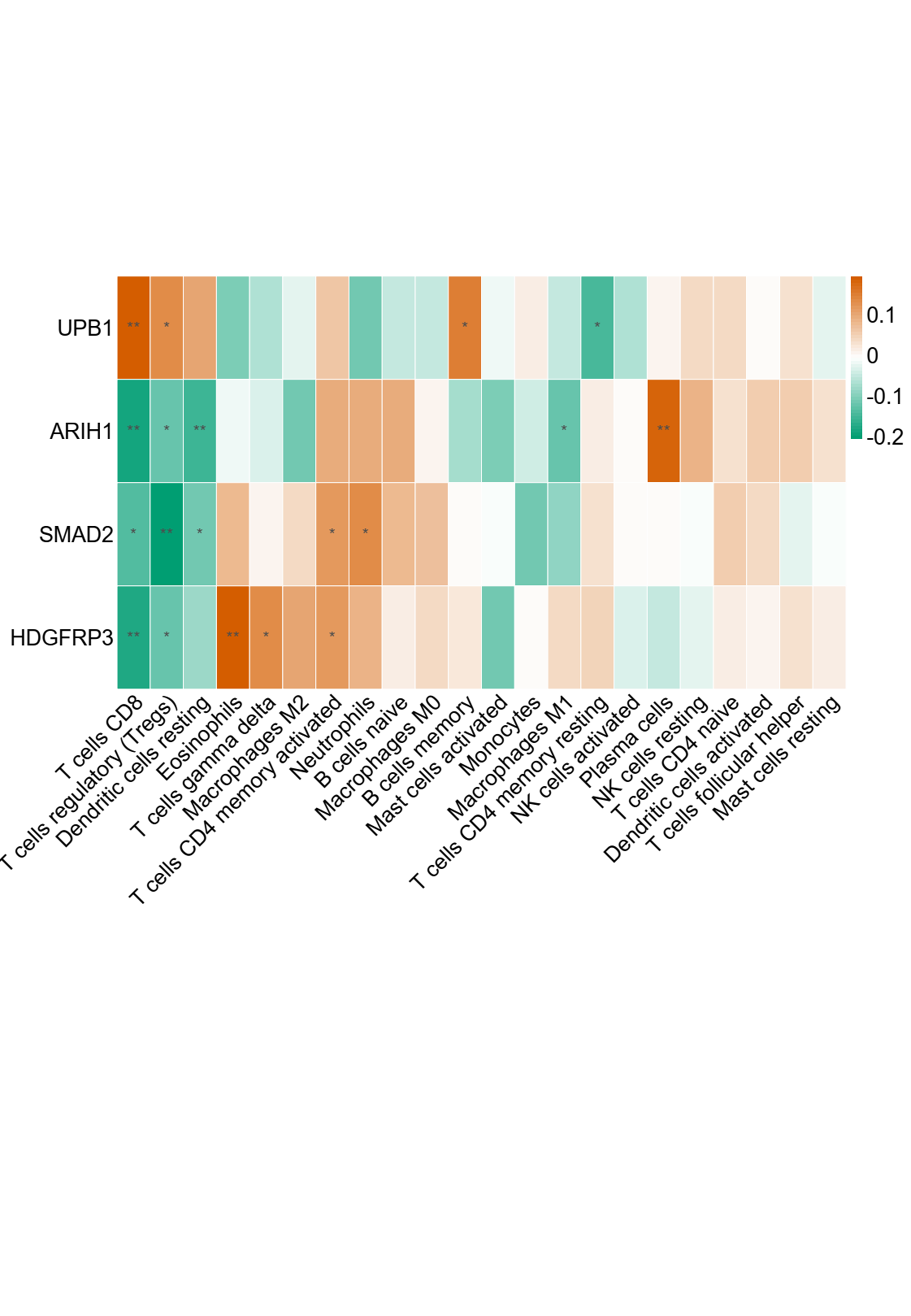

Supplement: Supplementary Figure 10 — Correlation of genes included in the model with the Tumor-Infiltrating Immune Cells. [file Image_10.tif]
