# Supplementary material for: A robust CD8+ T cell-related classifier for predicting the prognosis and efficacy of immunotherapy in stage III lung adenocarcinoma
Source: Front Immunol. 2022 Aug 31;13:993187. doi: 10.3389/fimmu.2022.993187 (PMC9471021; doi:10.3389/fimmu.2022.993187)
Supplement: Supplementary file 11 [file Table_1.docx]

Table S1 The samples were collected from 12 datasets included in the study.

| Series ID | No. of stage III LUAD patients | Platform | | Source |
| --- | --- | --- | --- | --- |
| TCGA_LUAD | 74 | Illumina RNAseq | | TCGA |
| GSE72094_GPL15048 | 57 | Rosetta/Merck Human RSTA Custom Affymetrix 2.0 microarray | | GEO |
| GSE30219_GPL570 | 2 | Affymetrix Human Genome U133 Plus 2.0 Array | | GEO |
| GSE37745_GPL570 | 13 | Affymetrix Human Genome U133 Plus 2.0 Array | | GEO |
| GSE29013_GPL570 | 8 | Affymetrix Human Genome U133 Plus 2.0 Array | | GEO |
| E-MTAB-923_GPL570 | 27 | Affymetrix Human Genome U133 Plus 2.0 Array | | ArrayExpress |
| GSE42127_GPL6884 | 20 | Illumina HumanWG-6 v3.0 expression beadchip | | GEO |
| GSE13213_GPL6480 | 25 | Agilent-014850 Whole Human Genome Microarray 4x44K G4112F | | GEO |
| GSE11969_GPL7015 | 25 | Agilent Homo sapiens 21.6K custom array | | GEO |
| GSE68465_GPL96 | 61 | Affymetrix Human Genome U133A Array | | GEO |
| GSE31547_GPL96 | 2 | Affymetrix Human Genome U133A Array | | GEO |
| GSE41271_GPL6884 | 48 | Illumina HumanWG-6 v3.0 expression beadchip | | GEO |
| Total patients | 362 |  |  |  |

Note: TCGA: (https://portal.gdc.cancer.gov/), GEO: (https://www.ncbi.nlm.nih.gov/geo/); ArrayExpress (https://www.ebi.ac.uk/arrayexpress/).
