# Supplementary material for: A robust CD8+ T cell-related classifier for predicting the prognosis and efficacy of immunotherapy in stage III lung adenocarcinoma
Source: Front Immunol. 2022 Aug 31;13:993187. doi: 10.3389/fimmu.2022.993187 (PMC9471021; doi:10.3389/fimmu.2022.993187)
Supplement: Supplementary file 12 [file Table_2.docx]

Table S2 Baseline clinicopathological characteristics of patients in the real-world validation cohort.

| Variables | All(%) | Group (%) | | *P value* |
| --- | --- | --- | --- | --- |
|  |  |  |  |  |
|  |  | high-risk | low-risk |  |
| ALL | 28(100) | 19(67.9) | 9(32.1) |  |
| Age(years) |  |  |  |  |
| ＜65 | 19(67.9) | 13(46.4) | 6(21.4) |  |
| ≥65 | 9(32.1) | 6(21.4) | 3(10.7) | 1.00 |
| Sex |  |  |  |  |
| Female | 16(57.1) | 11(39.3) | 5(17.9) |  |
| Male | 12(42.9) | 8(28.6) | 4(14.3) | 1.00 |
| Smoking history |  |  |  |  |
| Never | 18(64.3) | 12(42.9) | 6(21.4) |  |
| Former/Current | 10(35.7) | 7(25.0) | 3(10.7) | 1.00 |
| Laterality |  |  |  |  |
| Left | 12(42.9) | 8(28.6) | 4(14.3) |  |
| Right | 16(57.1) | 11(39.3) | 5(17.9) | 1.00 |
| Tumor size(cm) |  |  |  |  |
| ≤3 | 6(21.4) | 2(7.1) | 4(14.3) |  |
| >3 and ≤5 | 17(60.7) | 11(39.3) | 6(21.4) |  |
| >5 and ≤7 | 5(17.9) | 2(7.1) | 3(10.7) | 0.33 |
| >7 | 0(0) | 0(0) | 0(0) |  |
| N stage(8th) |  |  |  |  |
| N0 | 0(0) | 0(0) | 0(0) |  |
| N1 | 5(17.9) | 2(7.1) | 3(10.7) |  |
| N2 | 22(78.6) | 12(42.9) | 10(35.7) |  |
| N3 | 1(3.6) | 1(3.6) | 0(0) | 0.53 |
| PD-L1 expression, % |  |  |  |  |
| ＜50 | 4(14.3) | 3(10.7) | 1(3.6) |  |
| ≥50 | 9(32.1) | 6(21.4) | 3(10.7) |  |
| Not available | 15(53.6) | 10(35.7) | 5(17.9) | 0.94 |
| Type of treatment |  |  |  |  |
| Immunotherapy | 11(39.3) | 8(28.6) | 3(10.7) |  |
| Immunotherapy+Chemo | 17(60.7) | 11(39.3) | 6(21.4) | 1.00 |
