# Supplementary material for: A robust CD8+ T cell-related classifier for predicting the prognosis and efficacy of immunotherapy in stage III lung adenocarcinoma
Source: Front Immunol. 2022 Aug 31;13:993187. doi: 10.3389/fimmu.2022.993187 (PMC9471021; doi:10.3389/fimmu.2022.993187)
Supplement: Supplementary file 13 [file Table_3.docx]

Table S3 Differences in recent efficacy between high- and low-risk groups in the real-world cohort.

| Variables | All(%) | Group (%) | | *P value* |
| --- | --- | --- | --- | --- |
|  |  |  |  |  |
|  |  | high-risk | low-risk |  |
| Best overall response |  |  |  |  |
| CR | 3(10.7) | 2(7.1) | 1(3.6) |  |
| PR | 11(39.3) | 10(35.7) | 1(3.6) |  |
| ORR=CR+PR | 14(50.0) | 12(42.8) | 2(7.2) | 0.043 |
| SD | 12(42.8) | 6(21.4) | 6(21.4) |  |
| DCR=CR+PR+SD | 26(92.8) | 18(64.3) | 8(28.5) | 0.574 |
| PD | 2(7.2) | 1(3.6) | 1(3.6) |  |
